# Progress on lead-free metal halide perovskites for photovoltaic applications: a review

**DOI:** 10.1007/s00706-017-1933-9

**Published:** 2017-03-08

**Authors:** Sebastian F. Hoefler, Gregor Trimmel, Thomas Rath

**Affiliations:** Institute for Chemistry and Technology of Materials (ICTM), NAWI Graz, Graz University of Technology, Stremayrgasse 9, 8010 Graz, Austria

**Keywords:** Material science, Hybrid organic–inorganic materials, Solar cell, Transition metals compounds, Semiconductor

## Abstract

**Abstract:**

Metal halide perovskites have revolutionized the field of solution-processable photovoltaics. Within just a few years, the power conversion efficiencies of perovskite-based solar cells have been improved significantly to over 20%, which makes them now already comparably efficient to silicon-based photovoltaics. This breakthrough in solution-based photovoltaics, however, has the drawback that these high efficiencies can only be obtained with lead-based perovskites and this will arguably be a substantial hurdle for various applications of perovskite-based photovoltaics and their acceptance in society, even though the amounts of lead in the solar cells are low. This fact opened up a new research field on lead-free metal halide perovskites, which is currently remarkably vivid. We took this as incentive to review this emerging research field and discuss possible alternative elements to replace lead in metal halide perovskites and the properties of the corresponding perovskite materials based on recent theoretical and experimental studies. Up to now, tin-based perovskites turned out to be most promising in terms of power conversion efficiency; however, also the toxicity of these tin-based perovskites is argued. In the focus of the research community are other elements as well including germanium, copper, antimony, or bismuth, and the corresponding perovskite compounds are already showing promising properties.

**Graphical abstract:**

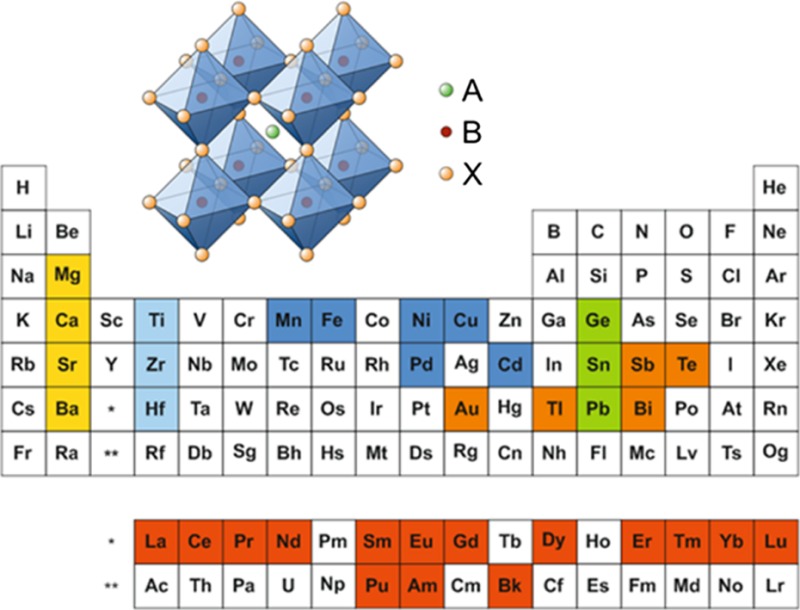

## Introduction

Perovskite-based solar cells employing metal halide perovskites as absorber materials belong to one of the most promising photovoltaic technologies for next-generation solar cells. This is illustrated by the remarkable increase in the power conversion efficiency (PCE) from 3.8% in 2009 [1] to now over 22% within a few years [2–4]. This outstanding performance is based on the exceptional properties of metal halide perovskites exhibiting high charge carrier mobilities, a balanced electron and hole transport, high absorption coefficients, direct and tunable band gaps [5], and long carrier diffusion lengths [6–8].

Another important advantage is that they can be prepared via a variety of different processing technologies, i.e. solution and vacuum-based techniques, and especially the facile low-temperature solution processability makes metal halide perovskite semiconductors that interesting [9–15]. Based on these assets, metal halide perovskites can already be regarded as a potential low-cost alternative to silicon-based photovoltaics.

The most extensively studied and also most efficient perovskite absorber materials are based on semiconducting (hybrid) lead halide perovskites adopting an ABX_3_ structure, where A is a monovalent organic cation (e.g. methylammonium (CH_3_NH_3_^+^, MA^+^), formamidinium (CH(NH_2_)_2_^+^, FA^+^) or an inorganic cation (e.g. K^+^, Rb^+^, Cs^+^), B is a divalent Pb^2+^ metal cation and the X-site of the perovskite structure is occupied by halide counterions (X = Cl^−^, Br^−^, I^−^). The properties of lead perovskites can be tuned by changing A-site or X-site ions and also mixed ion approaches turned out to be beneficial for the performance of the perovskites in photovoltaic devices.

Current limitations impeding the commercialization of lead-based halide perovskite solar cells are (1) the toxicity, bioavailability, and probable carcinogenicity of lead and lead halides, (2) the water solubility of lead that might contaminate water supplies, and (3) the chemical instability under ambient conditions, especially in the presence of air, humidity, and/or light [16–19].

These shortcomings are currently tackled by huge research efforts and progress could already be made in these fields. The stability of perovskite solar cells could be improved very recently by the partly exchange of the CH_3_NH_3_^+^ cation with CH(NH_2_)_2_^+^ and Cs^+^ ions in the triple cation approach [20] or by the addition of Rb^+^ as A-site cation [21]. These changes in the composition of the perovskite led to stable solar cells, which only lost 5% of their initial PCE within a 500-h test under illumination and maximum power point tracking [21].

The toxicity issue of lead halide perovskites is, however, still an unsolved drawback. Even though only low amounts are implemented in solar cells, there is a potential risk of harms on humans and environment [17, 18, 22–24].

Therefore, many research groups took up the challenge to substitute lead with other elements to find new non-toxic and environmentally benign perovskite materials suitable as efficient solar cell absorbers [25, 26]. Because of the fact that the perovskite crystal structure can be found in many compounds, many different material combinations are possible. However, due to these manifold possibilities, a huge number of materials needs to be screened. Table 1 shows an overview of the efficiencies of the currently best alternative lead-free halide perovskite materials and based on these PCE values, it is obvious that they currently cannot compete with lead-based materials, as today, the highest efficiencies for lead-free materials are about 6.4% for tin-based perovskites [27].

**Table 1:** Dimensionality, optical band gap, and power conversion efficiencies (PCEs) of the currently most promising lead-free perovskite absorber materials for photovoltaic applications

Perovskite	Dimensionality	Band gap/eV	PCE/%	References
CH_3_NH_3_SnI_3_	3D	1.23	6.4	[27]
CH_3_NH_3_GeI_3_	3D	2.0	0.20	[33]
(CH_3_(CH_2_)_3_NH_3_)_2_CuBr_4_	2D	1.76	0.63	[34]
Rb_3_Sb_2_I_9_	2D	2.1	0.66	[35]
Cs_3_Bi_2_I_9_	0D dimer	2.2	1.09	[36]

Perovskite-based solar cells are primarily prepared in two device architectures, one has been adopted from dye-sensitized solar cells using mainly mesoporous TiO_2_ as electron transport material and Spiro-OMeTAD (2,2′,7,7′-tetrakis[*N*,*N*-di(4-methoxyphenyl)amino]-9,9′-spirobifluorene) as hole transport material. The other one is derived from organic solar cells where PEDOT:PSS (poly(3,4-ethylenedioxythiophene)-poly(styrenesulfonate)) and PCBM ([6,6]-phenyl-C_61_-butyric acid methyl ester) are applied as hole and electron transport layer, respectively. Details to these device architectures and their influences on the performance of perovskite solar cells are described in recent reviews [28–32].

Currently, many research projects are initiated to identify further possible lead-free perovskite absorber materials and to incorporate them into tailored device architectures, giving rise to significant advancements in PCE of lead-free perovskite solar cells in the near future.

This review will focus on the class of lead-free metal halide perovskites for photovoltaic applications. It involves the results from experimental studies on lead-free metal halide perovskites and discusses insights from theoretical work for potential candidates to replace lead via both homo- and heterovalent substitution. Furthermore, we give a brief overview on lead-free metal chalcogenide perovskites, which also exhibit interesting properties for solar cell applications.

## Formability and structural considerations of perovskites

Perovskites are crystalline materials with an ABX_3_ structure similar to CaTiO_3_. Depending on the nature of the anionic species (X), oxide (O^2−^) and non-oxide perovskites such as chalcogenide (S^2−^, Se^2−^, Te^2−^) and halide (Cl^−^, Br^−^, I^−^) metal perovskites are distinguished. Moreover, molecular anions such as HCOO^−^ [37], BF_4_^−^ [38, 39], PF_6_^−^ [39], or SCN^−^ [40] were successfully incorporated as counterion.

In metal halide perovskites, the A-site is occupied by a monovalent organic (e.g. CH_3_NH_3_^+^, CH(NH_2_)_2_^+^ (NH_2_)_3_C^+^) or inorganic (e.g. K^+^, Rb^+^, Cs^+^) cation, the B-site by a divalent metal cation and the X-site by a halide counterion (Cl^−^, Br^−^, I^−^). Depending on the nature of the ions within the perovskite structure, hybrid organic–inorganic or purely inorganic metal halide perovskites are distinguished. A range of different divalent metal cations such as Pb^2+^, Sn^2+^, Ge^2+^, Mg^2+^, Ca^2+^, Sr^2+^, Ba^2+^, Cu^2+^, Fe^2+^, Pd^2+^, and Eu^2+^ have already been investigated as B-site cation.

**Fig. 1:**
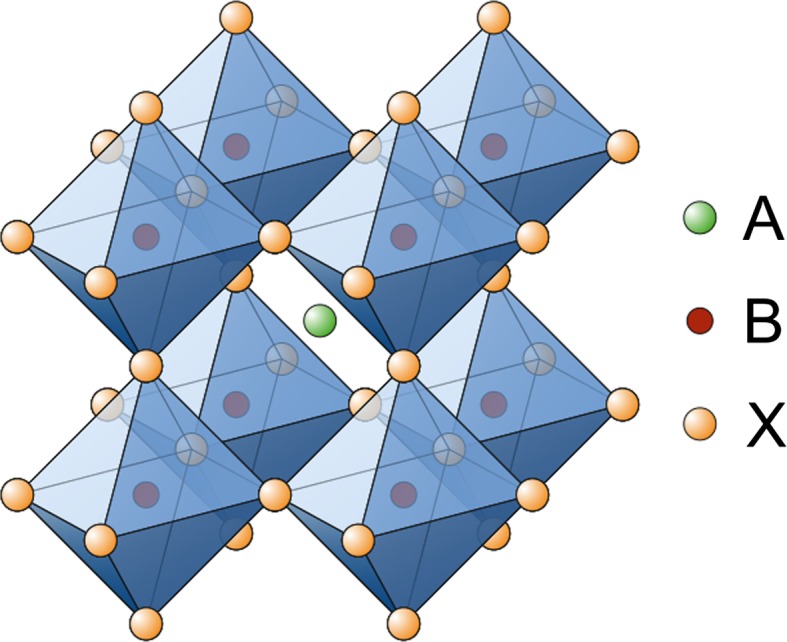
Crystal structure of ABX_3_-type metal halide perovskites

The ABX_3_-type perovskite structure consists of corner-sharing BX_6_ octahedra to form a three-dimensional network, whereby the A-site cations occupy the 12-fold coordinated (cuboctahedral) voids to maintain charge neutrality (Fig. 1). Alternatively, the perovskite structure can be described by a cubic close packed AX_3_ sublattice with divalent B-site cations within the sixfold coordinated (octahedral) cavities [41].

The formability of metal halide perovskites depends on various requirements: (1) charge neutrality between cations and anions, i.e. N(A) + N(B) = 3 N(X), whereby N represents the valence of the respective A, B, or X ions [42]; (2) the stability of the BX_6_ octahedra, which can be predicted by the octahedral factor *µ*; (3) the ionic radii of A, B, and X need to meet the requirements for the Goldschmidt tolerance factor *t* [43].

The octahedral factor *µ*, which is the ratio of the radii of the B-site cation (*r*_*B*_) and the halide counterion (*r*_*X*_), can be used to estimate the stability of the BX_6_ octahedra (Eq. 1) [41, 44]. The incorporation of the B-site cation is limited by ionic size restrictions defined by the X_6_ octahedron. For *µ* values between 0.442 and 0.895, metal halide perovskites have been found to be stable [45].

The Goldschmidt tolerance factor *t* is calculated according to Eq. (1) using the ionic radii of the involved A, B, and X ions (*r*_*A*_, *r*_*B*_, and *r*_*X*_) [41, 43, 44]. It can be used to evaluate which mismatches in size of the A, B, and X ions are tolerated to form perovskite-like structures:

1 $$\mu = \frac{{ r_{B} }}{{r_{X} }}\quad\quad t = \frac{{(r_{A} + r_{X} )}}{{\sqrt {2 } \left( {r_{B} + r_{X} } \right)}}.$$

Based on these ionic size restrictions for the involved cations and anions, a stability and formability range for ABX_3_ perovskite-like structures can be derived for which the tolerance factor was empirically found to be 0.8 ≤ *t* ≤ 1.0 [41]. A tolerance factor of 1.0, for example, indicates the formation of an ideal ABX_3_-type perovskite with a cubic crystal structure (e.g. SrTiO_3_ [46]). If the values for the tolerance factor are between 1.0 and 0.9, perovskites with a cubic crystal structure are formed predominantly. If the tolerance factor is lower (*t* = 0.80–0.89), distorted perovskite structures with orthorhombic, tetragonal, or rhombohedral crystal structures are more likely to be formed. If *t* < 0.8, the A cation is too small for the formation of a perovskite structure and, therefore, alternative structures such as the ilmenite-type FeTiO_3_ are formed instead. If *t* > 1.0, the A cation is too large for the formation of a perovskite structure. Hexagonal structures are introduced instead comprising layers of face-sharing octahedra [41, 47, 48].

The Goldschmidt tolerance factor concept was recently adapted for the family of hybrid organic–inorganic metal halide perovskite materials taking organic molecular cations such as CH_3_NH_3_^+^ into consideration [33, 47–50]. Moreover, these replacement rules are a viable tool to explain the concept of homovalent (isovalent) and heterovalent (aliovalent) substitution in metal halide perovskites. Therefore, the Goldschmidt replacement rules have attracted considerable attention recently to predict novel lead-free perovskite compounds for photovoltaic applications based on the ionic radii of the involved ions (see Table 2 for the radii of commonly used ions). Thereby, it is an essential concept that allows predictions for potential replacement candidates not only on the B-site but also on the other ion positions in the perovskite structure. The viability of this approach is shown by Kieslich et al., who theoretically studied divalent metal cations for homovalent substitution of lead in the perovskite structure to form hybrid metal halide perovskites via tolerance factor calculations [50]. Around 600 hypothetical perovskites were predicted as potential candidates that have not been reported yet including alkaline-earth metal- and lanthanide-based materials [50]. In addition, the tolerance factor concept was used to predict novel metal halide perovskites in various other investigations [41, 47–49].

**Table 2:** Effective ionic radii of organic molecular cations and Shannon ionic radii of inorganic cations as well as effective ionic radii of various anions [28, 48, 50, 51, 52]

Cation A	Effective radius *r*_*A*,eff_/pm	References	Cation B	Effective radius *r*_*B*,eff_/pm	References	Anion X	Effective radius *r*_*X*,eff_/pm	References
Ammonium, [NH_4_]^+^	146	[48]	Pb^2+^	119	[51]	Fluoride, F^−^	129	[28]
Hydroxylammonium, [NH_3_OH]^+^	216	[48]	Sn^2+^	110	[52]	Chloride, Cl^−^	181	[28]
Methylammonium, [CH_3_NH_3_]^+^	217	[48]	Sn^4+^	69	[51]	Bromide, Br^−^	196	[28]
Hydrazinium, [NH_3_NH_2_]^+^	217	[48]	Ge^2+^	73	[51]	Iodide, I^−^	220	[48]
Azetidinium, [(CH_2_)_3_NH_2_]^+^	250	[48]	Mg^2+^	72	[51]	Formate, HCOO^−^	136	[28]
Formamidinium, [CH(NH_2_)_2_]^+^	253	[48]	Ca^2+^	100	[51]			
Imidazolium, [C_3_N_2_H_5_]^+^	258	[48]	Sr^2+^	118	[51]			
Dimethylammonium, [(CH_3_)_2_NH_2_]^+^	272	[48]	Ba^2+^	135	[51]			
Ethylammonium, [(CH_3_CH_2_)NH_3_]^+^	274	[48]	Cu^2+^	73	[51]			
Guanidinium, [(NH_2_)_3_C]^+^	278	[48]	Fe^2+^	78	[51]			
Tetramethylammonium, [(CH_3_)_4_N]^+^	292	[48]	Pd^2+^	86	[51]			
Thiazolium, [C_3_H_4_NS]^+^	320	[50]	Eu^2+^	117	[51]			
3-Pyrrolinium, [NC_4_H_8_]^+^	272	[50]	Tm^2+^	103	[51]			
Tropylium, [C_7_H_7_]^+^	333	[50]	Yb^2+^	102	[51]			
Piperazinium, [C_4_H_12_N_2_]^2+^	322	[28]	Tl^+^	150	[51]			
Dabconium, [C_6_H_14_N_2_]^2+^	339	[28]	Au^+^	137	[51]			
K^+^	164	[51]	Au^3+^	85	[51]			
Rb^+^	172	[51]	Sb^3+^	76	[51]			
Cs^+^	188	[51]	Bi^3+^	103	[51]			
			Te^4+^	97	[51]			
			La^3+^	103	[51]			
			Ce^3+^	101	[51]			
			Pr^3+^	99	[51]			
			Nd^3+^	98	[51]			
			Sm^3+^	96	[51]			
			Eu^3+^	95	[51]			
			Gd^3+^	94	[51]			
			Dy^3+^	91	[51]			
			Er^3+^	89	[51]			
			Tm^3+^	88	[51]			
			Lu^3+^	86	[51]			
			Pu^3+^	100	[51]			
			Am^3+^	98	[51]			
			Bk^3+^	96	[51]			

Shannon ionic radii of metal cations consider the respective coordination sphere of the metal, i.e. sixfold (octahedral) coordination for alkali metals (K^+^, Rb^+^, Cs^+^) or 12-fold (cuboctahedral) coordination for the other ones

**Fig. 2:**
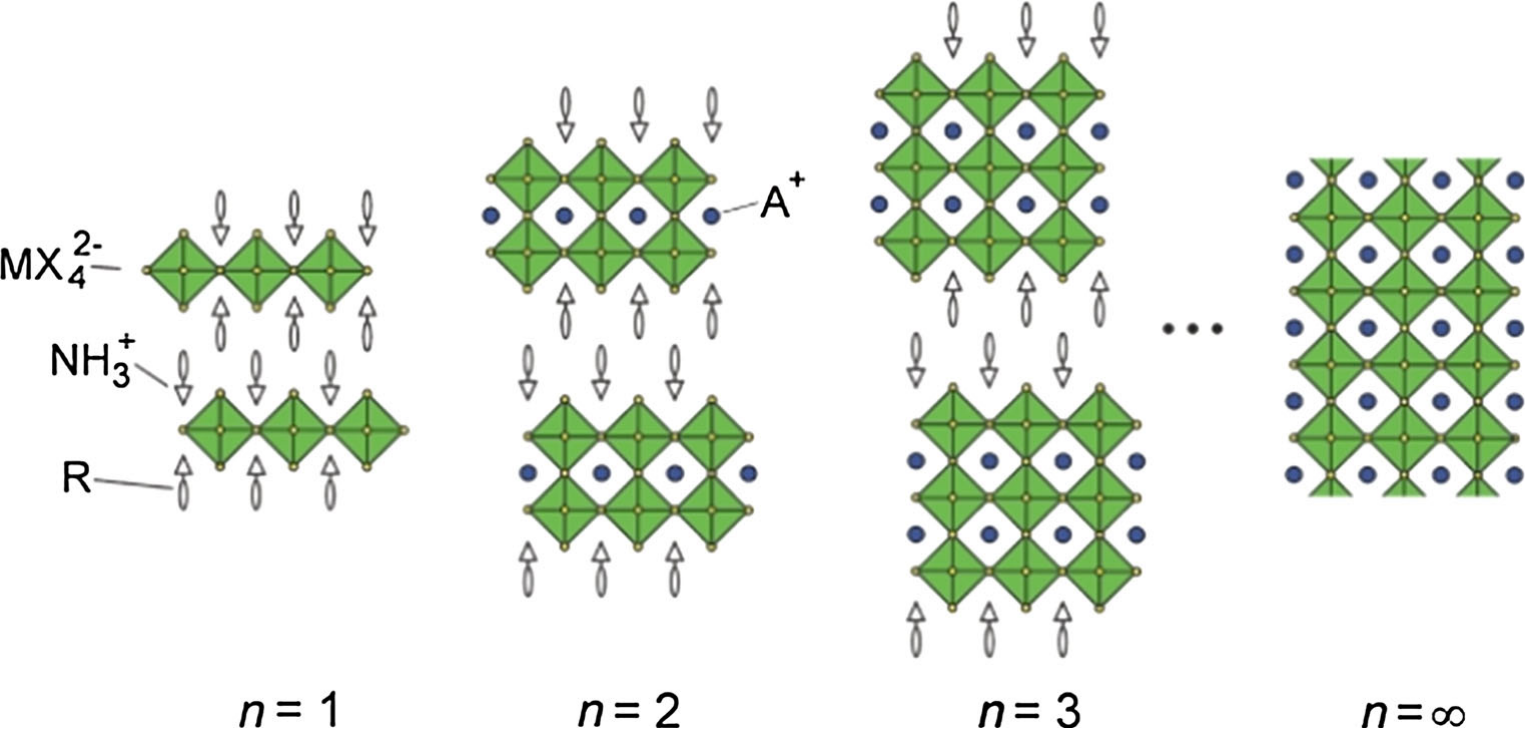
Schematic representation of the stacking of inorganic octahedral layers (*n*) in the 〈100〉-oriented two-dimensional perovskite structure. A three-dimensional perovskite is formed, when *n* is ∞. Reprinted with permission from [56]. Copyright (2001) Royal Society of Chemistry

Beyond the stability range of the Goldschmidt tolerance factor, perovskite-like derivatives with lower dimensionality can be found. For example, two-dimensional layered perovskites isostructural to Ruddlesden–Popper phases (e.g. (CH_3_NH_3_)_2_CuCl_*x*_Br_4−*x*_ [53]) are obtained by introducing large (interlayer) A-site cations (Fig. 2). However, for lower dimensional variants such as one-dimensional chain-like (e.g. HDABiI_5_, with HDA = 1,6-hexanediammonium ([H_3_NC_6_H_12_NH_3_]^2+^) [54]) or zero-dimensional structures (e.g. (CH_3_NH_3_)_3_Sb_2_I_9_ [55]), the Goldschmidt tolerance factor concept cannot be assessed in the same way since the aforementioned ionic size restrictions are gradually lifted [28].

A substitution of lead with nontoxic and environmentally benign elements forming lead-free metal halide perovskites can be generally achieved via two approaches:

1. homovalent substitution of lead with isovalent cations such as group-14 elements (Ge, Sn), alkaline-earth metals (Mg, Ca, Sr, Ba), transitions metals (Mn, Fe, Ni, Pd, Cu, Cd), and lanthanides (Eu, Tm, Yb),
2. heterovalent substitution with aliovalent metal cations such as transition metals (Au), main group elements (Tl, Sb, Bi, Te), lanthanides (La, Ce, Pr, Nd, Sm, Eu, Gd, Dy, Er, Tm, Lu), and actinides (Pu, Am, Bk). Since charge neutrality cannot be obtained with these ions in an ABX_3_ structure, a direct substitution is not possible in this case. However, a successful replacement of the divalent lead cation can be accomplished via a mixed-valence approach, i.e. an equal proportion of mono- and trivalent metal cations to give an overall divalent state in average to balance the total charge and valence [57], as reported for thallium [58, 59] and gold halide perovskites [60–62]. In addition, double halide perovskites (A_2_B^I^B^II^X_6_), which are based on the mixture of different mono- and trivalent metal cations, are a further approach towards heterovalent substitution [16, 63, 64]. Another possible avenue is based on the mixture of higher valent metal cations and vacancies to accommodate the total charge neutrality, which is accompanied by a considerable change in structure leading to A_3_B_2_X_9_-type perovskites (B = Sb, Bi) [35, 36, 55, 57, 65, 66]. However, these substitution approaches cannot be predicted via Goldschmidt replacement rules.

**Fig. 3:**
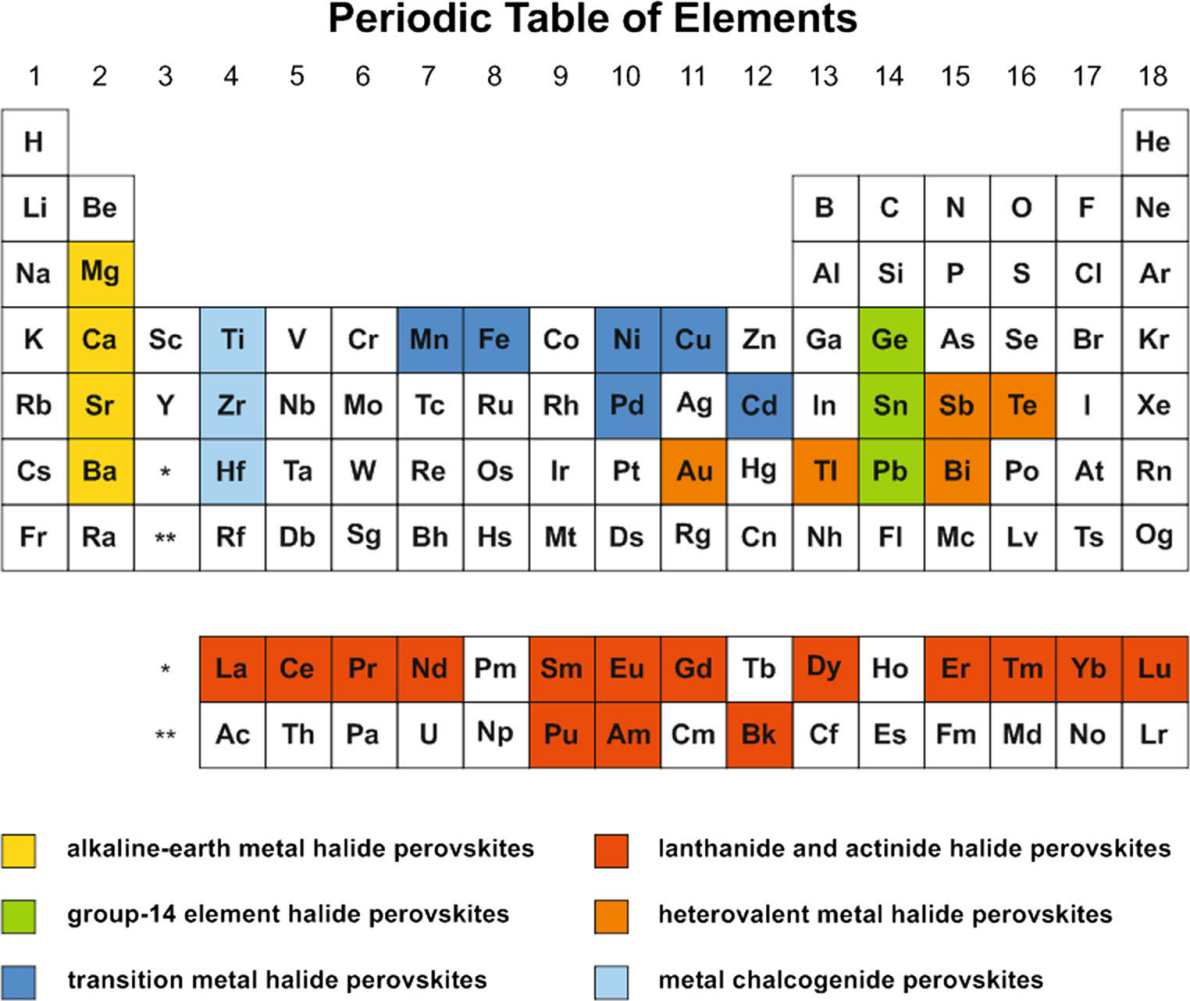
Lead replacement candidates in perovskite-type compounds from the periodic table of elements with the focus on homovalent substitution with group-14 elements (Ge, Sn), alkaline-earth metals (Mg, Ca, Sr, Ba), transition metals (Cu, Fe, Pd), lanthanides and actinides (Eu, Tm, Yb), heterovalent substitution with Tl, Au, Sb, Bi, and Te, and metal chalcogenide perovskites (Ti, Zf, Hf)

Homovalent and heterovalent substitution approaches lead to a wide range of lead-free metal halide perovskite semiconductors based on various elements in the periodic table (see Fig. 3), which are discussed in the following chapters.

## Homovalent substitution with divalent cations

A wide range of elements with a stable oxidation state of +2 are in principle suitable for homovalent substitution of lead in the perovskite structure. In particular, group-14 elements (Ge^2+^, Sn^2+^) but also alkaline-earth metals (Be^2+^, Mg^2+^, Ca^2+^, Sr^2+^, Ba^2+^), transition metals (V^2+^, Mn^2+^, Fe^2+^, Co^2+^, Ni^2+^, Pd^2+^, Pt^2+^, Cu^2+^, Zn^2+^, Cd^2+^, Hg^2+^), lanthanides (Eu^2+^, Tm^2+^, Yb^2+^), and p-block elements (Ga^2+^, In^2+^) can be considered for alternative lead-free perovskites [49, 50, 67]. However, some of these candidates have to be excluded due to their limited ability to form perovskites, or are not well suited for photovoltaic applications because of too high band gaps (Be, Ca, Sr, Ba), their toxicity (Cd, Hg), radioactivity, or their instability of the +2 oxidation state. As a consequence, based on the aforementioned considerations and computational screening of homovalent substitution of lead in the cesium and methylammonium metal halide perovskite, the most promising candidates are Sn^2+^, Ge^2+^, Mg^2+^, V^2+^, Mn^2+^, Ni^2+^, Zn^2+^, and Co^2+^ [49, 67].

### Group-14 element halide perovskites

The group-14 elements tin and germanium are the first logical candidates for the homovalent substitution of lead [27, 33, 68], as Sn^2+^ and Ge^2+^ have a similar electronic configuration as Pb^2+^. While tin and germanium halide perovskites have also good optoelectronic properties, both Sn^2+^ and Ge^2+^ ions possess a drawback compared to Pb^2+^ because they can be easily oxidized to the oxidation state +4 [27], which has its origin in the reduced inert pair effect and is even more pronounced for Ge^2+^ than for Sn^2+^.

However, this stability issue of tin and germanium halide perovskites is currently in the research focus of many groups of the “perovskite community” and some approaches towards increasing the stability have already been reported.

#### Tin halide perovskites

Sn^2+^ metal cations are the most obvious substitute for Pb^2+^ in the perovskite structure because of the similar s^2^ valence electronic configuration to Pb^2+^ and the similar ionic radius (Pb^2+^: 119 pm, Sn^2+^: 110 pm [52]), which makes it possible to form a perovskite with a basic formula ASnX_3_ (X = halide) in analogy to lead compounds. Even though tin is often presented as non-toxic alternative to lead, the toxicity of tin-based perovskites can be argued as well [22].

The most studied tin halide perovskites are CH_3_NH_3_SnI_3_ and CH(NH_2_)_2_SnI_3_. In addition, in analogy to the lead halide perovskites, the structural properties of the tin-based perovskites, i.e. dimensionality and connectivity of the perovskite lattice [69, 70], can be greatly affected by the size and functionality of the A-site cation as well as by the used halide. Small monovalent A-site cations (e.g. CH_3_NH_3_^+^, CH(NH_2_)_2_^+^, Cs^+^) lead to the formation of three-dimensional structures, whereas larger ones (e.g. cyclobutylammonium, tropylium) cause a reduced dimensionality such as two-dimensional layered, one-dimensional chain-like, or zero-dimensional structures [69, 71, 72]. These compositional and structural changes affect the optical and electronic properties as well.

**Fig. 4:**
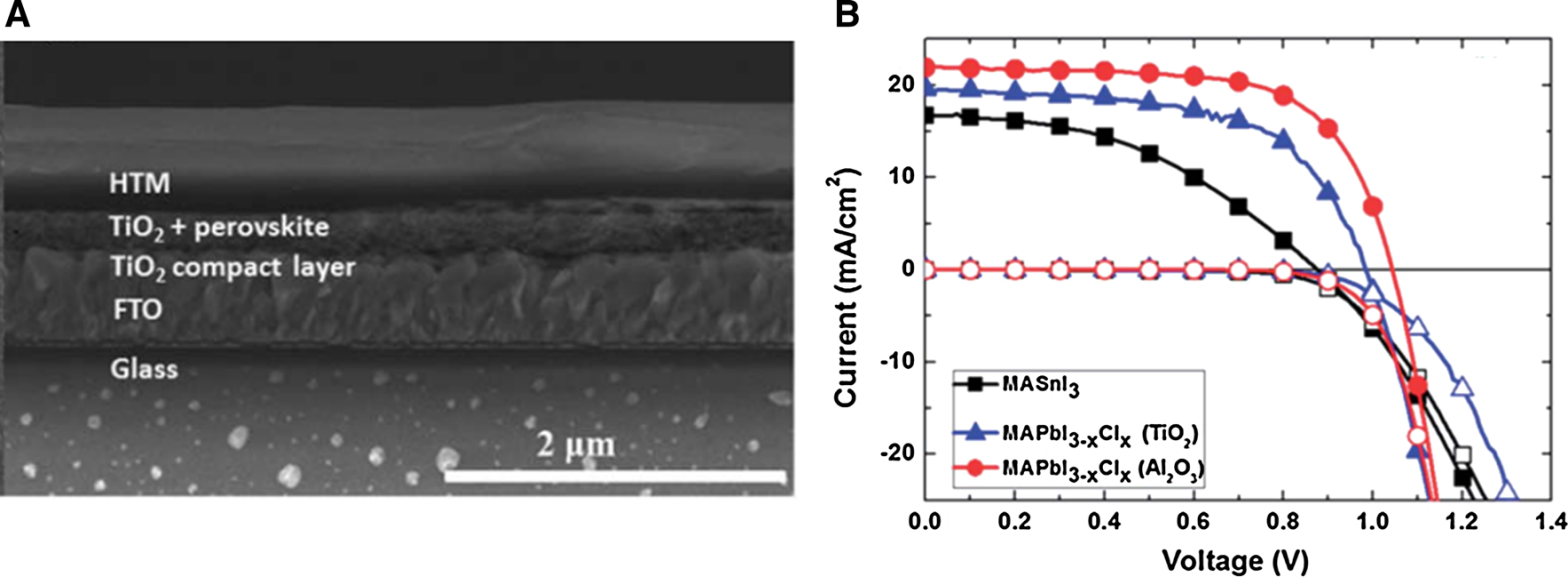
**a** Cross-sectional SEM image of a CH_3_NH_3_SnI_3_-based perovskite solar cell in meso-structured configuration. **b** *J*–*V* curves of tin- (CH_3_NH_3_SnI_3_) and lead-based (CH_3_NH_3_PbI_3−*x*_Cl_*x*_) perovskite solar cells under illuminated and dark conditions. Adapted with permission from [27]. Copyright (2014) Royal Society of Chemistry

The first study on an entirely lead-free tin halide perovskite semiconductor used as absorber material, namely methylammonium tin iodide (CH_3_NH_3_SnI_3_), was reported by Noel et al. [27]. The solar cells yielding PCE values over 6% were prepared in the device architecture glass/FTO/c-TiO_2_/mp-TiO_2_/CH_3_NH_3_SnI_3_/Spiro-OMeTAD/Au (FTO: fluorine-doped tin oxide, c: compact, mp: mesoporous). A scanning electron microscopy (SEM) image of the cross section of the corresponding device is shown in Fig. 4a. Using mesoporous TiO_2_ has been beneficial due to the shorter charge carrier diffusion lengths of the tin halide perovskite compared to the lead-based analogue. Because of the challenging stability of tin halide perovskites, solar cell preparation had to be performed entirely in inert atmosphere starting from highly pure precursor materials. It is also remarkable that an open-circuit voltage (*V*_OC_) of 0.88 V was obtained using an absorber material which has a relatively low band gap of 1.23 eV. The obtained short-circuit current density (*J*_SC_) was 16.8 mA cm^−2^ and the fill factor (FF) was 42% (Fig. 4b).

**Fig. 5:**
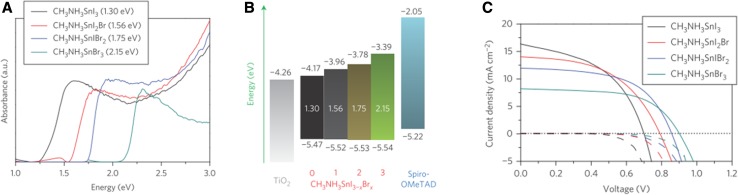
**a** Absorption properties, **b** energy level diagram and **c** *J*–*V* curves of CH_3_NH_3_Sn(I,Br)_3_-based perovskite solar cells. Adapted with permission from [75]. Copyright (2014) Macmillan Publishers Limited

By substituting the I^−^ counterion with other halides, a range of different tin halide perovskite analogues CH_3_NH_3_SnX_3_ (X = Cl, Br) is accessible with calculated band gaps in the range of 1.7–3.0 eV [73]. CH_3_NH_3_SnBr_3_, for example, with an optical band gap of ca. 2.2 eV can be processed via vapor deposition-based methods using SnBr_2_ and CH_3_NH_3_Br as starting compounds [74]. Jung et al. reported PCE values of 0.35% (co-evaporation) and 1.12% (sequential deposition) for planar perovskite solar cells with CH_3_NH_3_SnBr_3_ as absorber material [74]. The optical band gap can be further fine-tuned via the halide ratio using a mixed halide approach. By variation of the I:Br ratio, the optical band gap can be engineered between 1.3 eV (CH_3_NH_3_SnI_3_) and 2.15 eV (CH_3_NH_3_SnBr_3_) [75]. Based on this approach, Hao et al. reported a mixed iodide–bromide tin perovskite semiconductor (CH_3_NH_3_SnIBr_2_) with an optical band gap of 1.75 eV yielding a PCE of 5.73% in meso-structured perovskite solar cells [75]. Figure 5a shows the absorption properties of the mixed halide tin perovskites and Fig. 5b the corresponding energy levels of the compounds, which reveal almost no change in the valence band position and an upward shift of the conduction band position when increasing the bromide content. The *J*–*V* curves are presented in Fig. 5c showing the correlation of decreasing *J*_SC_ and increasing *V*_OC_ when the band gap of the respective perovskite material becomes wider.

By introducing the CH(NH_2_)_2_^+^ ion into tin iodide perovskites forming CH(NH_2_)_2_SnI_3_, the band gap is widened to 1.41 eV. CH(NH_2_)_2_SnI_3_ has, in contrast to CH(NH_2_)_2_PbI_3_, only one thermally accessible phase, which is stable up to 200 °C. By adding SnF_2_, which increases the stability of Sn^2+^, a PCE of 2.1% could be obtained [76, 77]. Further optimization using the SnF_2_–pyrazine complex causing a more homogeneous distribution of SnF_2_ in the perovskite led to PCE values of 4.8% [78] and recently the efficiency of this material could be further increased to 6.22% [79]. In this latter study, PEDOT:PSS and fullerene (C_60_) have been used as hole transport layer (HTL) and electron transport layer (ETL) in contrast to the both aforementioned reports, in which Spiro-OMeTAD and TiO_2_ have been used. Moreover, this study points out that similar to the lead-based perovskites solvent treatment during spin coating is crucial for the performance of tin-based perovskite solar cells and diethyl ether dripping was found to give the best results in terms of PCE and reproducibility. In a further study, mixed iodide/bromide CH(NH_2_)_2_Sn-halide perovskites led to a PCE of 1.72% using MoO_3_ as hole transport material [80].

Introducing Cs^+^ as cation leads to CsSnI_3_, which is thermally even more stable than CH(NH_2_)_2_SnI_3_ and melts at 451 °C [81, 82]. Two polymorphs are existing at room temperature: B-γ-CsSnI_3_, a black orthorhombic phase, suitable for solar cell applications [81, 83, 84], and a yellow Y-CsSnI_3_ phase with a one-dimensional double-chain structure [83, 85, 86]. B-γ-CsSnI_3_ has a direct band gap of 1.3 eV [82] and by preparing it via an alternating deposition of SnCl_2_ and CsI layers followed by a thermal treatment at 175 °C, solar cells with a PCE of 0.9% could be obtained in a glass/ITO/CsSnI_3_/Au/Ti device structure (ITO: indium tin oxide) [87]. By a controlled grain-coarsening of CsSnI_3_ films based on heat treatment and using a planar device architecture (NiO_*x*_ as HTL, PCBM as ETL) solar cells with a PCE of 3.31% have been reported by Wang et al. [88]. The addition of 20% SnF_2_ to CsSnI_3_ was found to positively influence the solar cell performance in meso-structured perovskite solar cells and a PCE of 2.02% was reached [89]. The addition of SnF_2_ lowers the background charge carrier density by neutralizing traps [89, 90].

CsSnBr_3_ possesses a direct band gap of 1.75 eV and solar cells with efficiencies of up to 2.1% have been reported using this material as absorber layer [90]. However, CsSnBr_3_-based solar cells currently suffer from a low *V*_OC_ (up to 420 mV) stemming most likely from a mismatch of the energy levels of the materials (TiO_2_, CsSnBr_3_, Spiro-OMeTAD) used in these devices, which gives space for further optimization by investigating better suited ETLs and HTLs. Mixed chloride/bromide cesium tin halide perovskites reveal PCE values of up to 3.2% as well as a good thermal and device stability [91, 92].

Because of its good p-type conductivity under Sn-poor conditions [93], CsSnI_3_ can be used as solution-processable HTL in solid-state dye-sensitized solar cells. By SnF_2_-doping forming CsSnI_2.95_F_0.05_, a PCE of 8.51% (using the dye N719 as sensitizer) could be obtained [82, 94]. This report considers the perovskite layer as HTL; however, based on the presented spectral response measurements of the solar cells, it seems that also the perovskite itself contributes to the overall PCE, and thus these solar cells should be seen more as mixed dye-sensitized/perovskite solar cells.

Even though encouraging stability data have already been reported, the main drawback of tin halide perovskites is still the chemical instability of the divalent metal cation, which is due to the oxidation of Sn^2+^ to Sn^4+^ in ambient conditions [27]. As a consequence, the oxidation of Sn^2+^ to the chemically more stable Sn^4+^ analogue impedes the charge neutrality of the perovskite and causes the degradation of the perovskite by formation of oxides/hydroxides of tin, and furthermore Sn^4+^ leads to hole doping of the material [27, 95]. To avoid oxidation, inert processing and rigorous encapsulation of the tin-based perovskite devices are necessary.

To overcome this oxidation stability issue, double perovskite semiconductors with a basic formula A_2_SnX_6_ (A = Cs, C_7_H_7_, X = halide) have been introduced [69, 96–99]. The double perovskite structure is built up from face-centered nearly isolated SnX_6_ octahedra, in which the cuboctahedral voids are occupied by A-site cations [96]. In this structure, tin has the more stable oxidation state +4 resulting in improved air and moisture stability and processability [69, 96–99]. Due to enhanced air stability and promising photovoltaic properties [100], tin-based double perovskite semiconductors (e.g. Cs_2_SnI_6_) have recently been considered as absorber material in perovskite solar cells yielding PCE values of almost 1% [99]. Alternatively, double perovskites were discussed as hole transport materials (Cs_2_SnI_6_ [97], Cs_2_SnI_3_Br_3_ [101]) in solid-state dye-sensitized solar cells using classical dyes as absorbers leading to PCE values up to 7.8% [97].

Furthermore, optoelectronically active cations like the tropylium (C_7_H_7_^+^) ion have been investigated as A-site cation in tin halide perovskites. (C_7_H_7_)_2_SnI_6_ appears as a deep black solid, and crystallizes in a monoclinic crystal system containing isolated tin(IV)-iodide octahedra [69].

A summary of structural and optical data of tin halide perovskites and their performance as absorber material in photovoltaic devices is given in Table 3.

**Table 3:** Structural and optical data of tin halide perovskites and the highest obtained PCEs (if applied in photovoltaic devices)

Perovskite	Sim./exp.	Crystal system (space group)	Dimensionality	Band gap/eV	PCE/%	References
CH_3_NH_3_SnBr_3_	Exp.	Pseudocubic (*P*4*mm*)	3D	2.15–2.2	4.27	[74, 75]
CH_3_NH_3_SnIBr_2_	Exp.	Pseudocubic (*P*4*mm*)	3D	1.75	5.73	[75]
CH_3_NH_3_SnI_2_Br	Exp.	Pseudocubic (*P*4*mm*)	3D	1.56	5.48	[73, 75]
CH_3_NH_3_SnI_3_	Exp.	Pseudocubic (*P*4*mm*)	3D	1.27–1.35	5.23	[75, 102, 103]
		Tetragonal (*P*4*mm*)		1.21–1.35	6.4	[27, 71]
CH(NH_2_)_2_SnI_2_Br	Exp.	Orthorhombic (–)	3D	1.68	1.72	[80]
CH(NH_2_)_2_SnI_3_	Exp.	Orthorhombic (*Amm*2)	3D	1.4–1.41	6.22	[71, 76, 78, 79]
(C_7_H_7_)_2_SnI_6_	Exp.	Monoclinic (–)	0D	1.2	–	[69]
CsSnCl_3_	Exp.	Monoclinic (–)	3D	2.8	–	[104]
CsSnBrCl_2_	Exp.	Monoclinic (–)	3D	2.1	–	[104]
CsSnBr_2_Cl	Exp.	Cubic (–)	3D	1.9	–	[104]
CsSnBr_3_	Exp.	Cubic (–)	3D	1.75–1.8	2.1	[90, 91, 104, 105]
CsSnIBr_2_	Exp.	Cubic (–)	3D	1.63–1.65	3.2	[91, 92, 104]
CsSnI_2_Br	Exp.	Cubic (–)	3D	1.37–1.41	1.67	[91, 104]
CsSnI_3_	Exp.	Orthorhombic (–)	3D	1.27–1.31	3.31	[82, 88, 89, 91, 104]
CsSnI_2.95_F_0.05_	Exp.	Orthorhombic (*Pnma*)	3D	1.3	8.51^a^	[82]
Cs_2_SnCl_6_	Exp.	Cubic ($Fm\bar{3}m$ *m*)	3D	3.9	0.07^a^	[106]
Cs_2_SnBr_6_	Exp.	Cubic ($Fm\bar{3}m$ *m*)	3D	2.7	0.04^a^	[106]
Cs_2_SnI_6_	Exp.	Cubic ($Fm\bar{3}m$ *m*)	3D	1.26–1.62	0.86, 7.8^a^	[97–99, 106]
Cs_2_SnI_3_Br_3_	Exp.	Cubic (*Fm* $\bar{3}$ *m*)	3D	1.43	3.63^a^	[101]

^a^Perovskite was used as HTL in a dye-sensitized solar cell

#### Germanium halide perovskites

Another potential candidate for the substitution of lead in the perovskite structure is the group-14 metalloid germanium. In comparison to Pb^2+^, the divalent germanium cation (Ge^2+^) is in the same oxidation state but exhibits a lower electronegativity, a more covalent character and an ionic radius (73 pm) lower than Pb^2+^ (119 pm) [51, 52]. Nevertheless, Goldschmidt tolerance factor calculations support the formation of germanium halide perovskites, as shown for CH_3_NH_3_GeX_3_ (X = Cl, Br, I) compounds with tolerance factor values of 1.005 (CH_3_NH_3_GeCl_3_), 0.988 (CH_3_NH_3_GeBr_3_), and 0.965 (CH_3_NH_3_GeI_3_), which coincide with *t* values reported for the ideal perovskite structure (0.97 < *t* < 1.03) [107, 108].

Theoretical considerations using density functional theory (DFT) methods show that germanium halide perovskites have high absorption coefficients as well as similar absorption spectra and carrier transport properties as the lead analogues [33, 42, 107, 109]. First-principle calculations of CsGeX_3_ (X = Cl, Br, I) perovskites show that the band gaps depend on the halide ion, i.e. CsGeCl_3_ (3.67 eV) > CsGeBr_3_ (2.32 eV) > CsGeI_3_ (1.53 eV) [108], see also Table 4. Moreover, mixed halide germanium perovskites such as Cs_2_GeCl_2_I_4_, Cs_2_GeBr_2_I_4_, and Cs_2_GeI_2_Br_4_ were predicted to be promising direct band gap semiconductors [109]. Sun et al. extended the theoretical investigations to hybrid germanium halide perovskites, namely to CH_3_NH_3_GeX_3_ (X = Cl, Br, I) compounds [107]. The calculated band gaps based on PBE (Perdew–Burke–Ernzerhof) functionals were found to show a similar trend as for the cesium-based compounds, i.e. CH_3_NH_3_GeCl_3_ (3.76 eV) > CH_3_NH_3_GeBr_3_ (2.81 eV) > CH_3_NH_3_GeI_3_ (1.61 eV) [107] and the band gaps of the iodide-based compounds are similar to the lead analogues CsPbI_3_ (1.73 eV) and CH_3_NH_3_PbI_3_ (1.57 eV) [110].

**Table 4:** Structural and optical data of germanium halide perovskites and the obtained PCEs (if used as absorber material in photovoltaic devices)

Perovskite	Sim./exp.	Crystal system (space group)	Dimensionality	Band gap/eV	PCE/%	References
RbGeCl_3_·x H_2_O	Exp.	Monoclinic (*P*2_1_/*m*)	3D	3.84	–	[114]
RbGeBr_3_	Exp.	Trigonal (*R*3*m*)	3D	2.74	–	[115]
(Rb_*x*_Cs_1−*x*_)GeBr_3_	Exp.	Trigonal (*R*3*m*)	3D	2.4 (*x* = 0.25)	–	[115]
				2.4 (*x* = 0.5)		
				2.4 (*x* = 0.75)		
CsGeCl_3_	Exp.	Trigonal (*R*3*m*)	3D	3.4–3.67	–	[108]
CsGeBr_3_	Exp.	Trigonal (*R*3*m*)	3D	2.32–2.4	–	[108]
CsGe(Br_*x*_Cl_1−*x*_)_3_	Exp.	Trigonal (*R*3*m*)	3D	2.65 (*x* = 0.25)	–	[115]
				2.5 (*x* = 0.5)		
				2.47 (*x* = 0.75)		
CsGeI_3_	Sim./exp.	Trigonal (*R*3*m*)	3D	1.53–1.63	0.11	[33, 42, 68, 108, 116, 117]
CH_3_NH_3_GeCl_3_	Sim.	Trigonal	3D	3.74–3.76	–	[107, 117]
CH_3_NH_3_GeBr_3_	Sim.	Trigonal	3D	2.76–2.81	–	[107, 117]
CH_3_NH_3_GeI_3_	Exp.	Trigonal (*R*3*m*)	3D	1.9–2.0	0.20	[33, 68, 117]
CH(NH_2_)_2_GeI_3_	Exp.	Trigonal (*R*3*m*)	3D	2.2–2.35	–	[33, 68, 117]
MFOGeI_3_	Exp.	Monoclinic (*P*2_1_)	3D	2.5	–	[68, 117]
GUAGeI_3_	Exp.	Monoclinic (*P*2_1_/*c*)	3D	2.7	–	[68, 117]
TMAGeI_3_	Exp.	Hexagonal (*P*6_3_)	3D	2.8	–	[68]
IPAGeI_3_	Exp.	Tetragonal (*I* $\bar{4}$2*d*)	3D	2.7	–	[68]

*MFO* acetamidinium, *GUA* guanidinium, *TMA* trimethylammonium, *IPA* isopropylammonium

Germanium halide perovskites, however, have rarely been investigated experimentally, which is presumably due to the chemical instability upon oxidation of the divalent Ge^2+^ cation [33, 68]. Due to the reduced inert electron pair effect, this oxidation stability issue is even more prominent in germanium-based perovskites than in tin-based ones.

Stoumpos et al. thoroughly investigated the structural, electronic and optical properties of germanium halide perovskites with the basic formula AGeI_3_ incorporating Cs^+^ and different organic A-site cations [68]. Depending on the A-site cation, different structures can be formed. Small cations such as Cs^+^, CH_3_NH_3_^+^ or CH(NH_2_)_2_^+^ ions form three-dimensional perovskite frameworks based on GeI_6_^4−^ corner-sharing octahedra [68]. Bigger A-site cations (e.g. guanidinium, trimethylammonium) lead to distortions of the crystal structure and one-dimensional chain-like hexagonal perovskite structures (CsCdBr_3_-type) consisting of GeI_6_^4−^ face-sharing octahedra are formed [33, 68]. Using the *n*-butylammonium ion as A-site cation, the orthorhombic perovskite (C_4_H_9_NH_3_)_2_GeI_4_ is formed exhibiting a two-dimensional structure in which perovskite sheets consisting of corner-sharing GeI_6_ octahedra are separated by bilayers of *n*-butylammonium cations [111].

The A-site cation of the perovskite structure is particularly important for band gap engineering [33, 68]. For AGeI_3_ with a three-dimensional structure, the band gap was found to systematically increase when replacing the small Cs^+^ cation (1.6 eV) with larger ones such as CH_3_NH_3_^+^ (1.9 eV), CH(NH_2_)_2_^+^ (2.2 eV), or acetamidinium (2.5 eV) [68]. Substitution with even bulkier organic cations does not only reduce the dimensionality of the perovskite framework but also further increases the band gap, e.g. trimethylammonium (2.8 eV), guanidinium (2.7 eV), and isopropylammonium (2.7 eV) [68]. Moreover, three-dimensional perovskites are materials with a direct band gap, while one-dimensional compounds exhibit indirect band gaps [68].

**Fig. 6:**
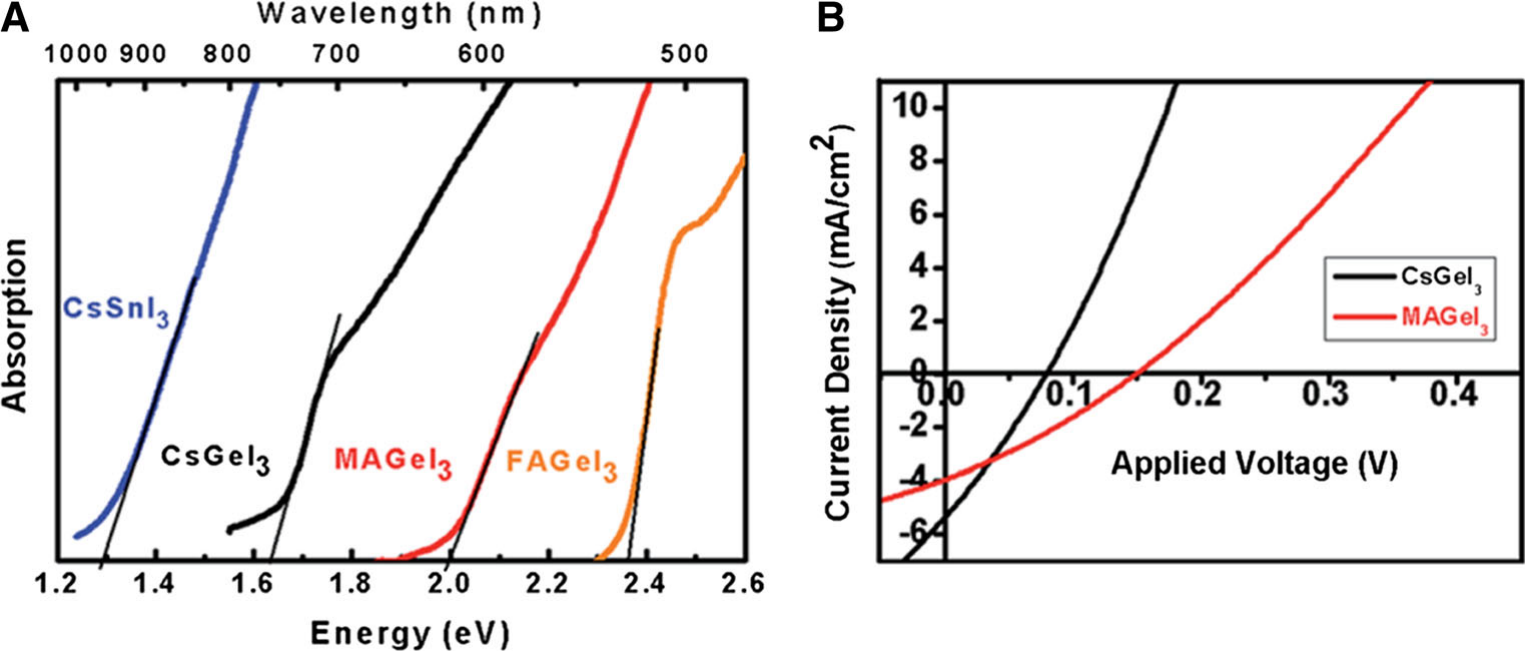
**a** UV–Vis absorption data of CsSnI_3_, CsGeI_3_, CH_3_NH_3_GeI_3_, and CH(NH_2_)_2_GeI_3_ and **b** *J*–*V* curves of CsGeI_3_- and CH_3_NH_3_GeI_3_-based solar cells. Adapted with permission from [33]. Copyright (2015) Royal Society of Chemistry

CsGeI_3_ and CH_3_NH_3_GeI_3_ have already been implemented as absorber materials in meso-structured perovskite solar cells yielding PCE values of 0.11 and 0.20%, respectively (Fig. 6) [33]. This moderate performance might be due to the oxidation of Ge^2+^ to Ge^4+^ already occurring during the fabrication of the solar cell [33] and the limited *V*_OC_, in particular of the CsGeI_3_ (74 mV), was suggested to originate from the defect chemistry in this material [112].

In a patent by Huang et al., a PCE of approximately 3% in a meso-structured perovskite solar cell architecture is claimed, however, with limited experimental data [113]. This value is still much lower compared to the theoretically possible PCE values of 27.9% predicted by Qian et al. and further effort has to be made to improve the efficiency of germanium-based perovskites to competitive values [42]. Nonetheless, germanium halide perovskites are promising low-temperature solution-processable semiconductors for photovoltaic applications and the full potential of this material is by far not exploited yet.

### Alkaline-earth metal halide perovskites

Alkaline-earth metals such as magnesium, calcium, strontium, and barium can be potential homovalent substitutes due to ionic radii suitable to form perovskite structures, a high abundance in the Earth’s crust, non-toxicity, and stable +2 oxidation states similar to Pb^2+^ [47, 118]. Alkaline-earth metal halide perovskites with a basic formula ABX_3_ (B = Mg, Ca, Sr; X = Cl, Br, I) employing inorganic A-site cations (e.g. K^+^, Cs^+^) have been studied extensively with regard to their photoluminescence properties resulting from doping with rare earth metal cations such as Eu^2+^, Yb^2+^, or Tm^2+^ [119–123]. Until now, only a few research studies have been focused on alkaline-earth-metal-based halide perovskites for photovoltaic applications, which is due to the high calculated band gaps and extreme sensitivity towards humidity [118].

#### Magnesium halide perovskites

According to simulations by Filip et al. and Choudhary et al., Mg^2+^ can replace lead in the perovskite structure forming magnesium halide perovskites with low effective masses, reasonable absorption coefficients, and direct band gaps tunable within the visible range of the electromagnetic spectrum depending on the size of the A-site cation [49, 67]. In case of AMgI_3_ perovskites, the band gap was predicted to be tunable using different A-site cations with calculated band gaps of 0.9 eV (CH(NH_2_)_2_MgI_3_), 1.5 eV (CH_3_NH_3_MgI_3_), and 1.7 eV (CsMgI_3_) [49]. Theoretical calculations predicted magnesium halide perovskites to be stable despite the smaller ionic radius of Mg^2+^ (72 pm) compared to Pb^2+^ (119 pm) [49, 51]. Suta et al. synthesized Eu^2+^-doped CsMgI_3_, which crystallizes in a CsNiCl_3_ structure (a distorted perovskite structure) comprising face-sharing MgI_6_^4−^ octahedra which feature linear chains along the *c*-axis and 12-fold coordinated Cs^+^ ions in the anti-cuboctahedral positions [121]. To our knowledge, magnesium halide perovskites have not been implemented as absorber materials in solar cells yet, which might be due to the sensitivity towards humidity [121].

#### Calcium halide perovskites

Calcium is a nontoxic, low-cost alkaline-earth metal with high abundance in the Earth’s crust. The divalent Ca^2+^ ion has an adequate ionic radius (100 pm) similar to Pb^2+^ (119 pm) capable to exchange lead in the perovskite structure [47, 51, 118].

Based on DFT calculations, Pazoki et al. predicted that CH_3_NH_3_CaI_3_ forms a stable perovskite structure with a calculated band gap of 2.95 eV, which is much higher compared to the lead analogue (1.66 eV) [118].

Uribe et al. synthesized CH_3_NH_3_CaI_3_ and CH_3_NH_3_CaI_3−*x*_Cl_*x*_ with pseudocubic structure via a low-temperature solution-based route from CH_3_NH_3_I mixed with CaI_2_ or CaCl_2_ as precursors [47]. The optical band gap of CH_3_NH_3_CaI_3_ was determined to be 3.78 eV matching quite well with the calculated band gap of 3.4 eV [47]. Due to the high band gap, the low mobility and the instability in moist atmosphere, calcium halide perovskites are not very suitable for photovoltaic applications but might be possible candidates for charge-selective contacts [47, 118].

#### Strontium halide perovskites

Strontium is a fairly nontoxic, relatively inexpensive, highly abundant alkaline-earth metal with an ionic radius (Sr^2+^: 118 pm) very similar to lead (Pb^2+^: 119 pm), which makes strontium a suitable candidate for homovalent substitution of lead in the perovskite without affecting the crystal structure [51, 124].

The current research in the field of strontium halide perovskites for optoelectronic applications is mainly focusing on CH_3_NH_3_SrI_3_ [118, 124]. DFT calculations of Jacobsson et al. and Pazoki et al. predict the formation of a stable CH_3_NH_3_SrI_3_ perovskite material despite the electronegativity difference between lead (2.33) and strontium (0.95) [118, 124, 125]. This lower electronegativity of strontium together with the missing d-orbitals in the valence of Sr^2+^ are responsible for a significantly higher band gap of 3.6 eV (CH_3_NH_3_SrI_3_) compared to the lead analogue (1.66 eV) [118, 124] and thus limit a possible application as absorber material in solar cells. In addition, the higher electronegativity difference between metal and halogen leads to more pronounced ionic interactions of the metal–halogen bond in strontium perovskites [118, 124]. CH_3_NH_3_SrI_3_ can be prepared following a one-step solution-based processing route from CH_3_NH_3_I and SrI_2_. Alternatively, vapor phase or layer-by-layer deposition methods are suggested as preparation pathways [124]. CH_3_NH_3_SrI_3_ exhibits a poor stability under ambient conditions due to its hygroscopic nature. Alternatively, Pazoki et al. suggested a potential application as charge-selective contact material [118].

#### Barium halide perovskites

The stable Ba^2+^ metal cation exhibits a slightly larger ionic radius (135 pm) compared to Pb^2+^ (119 pm) [51]. Applying the Goldschmidt replacement rules, CH_3_NH_3_BaI_3_ has a tolerance factor *t* of 0.797, which is similar to the lead halide perovskite analogue CH_3_NH_3_PbI_3_ (*t* = 0.83) [126]. Consequently, CH_3_NH_3_BaI_3_ is expected to have a similar crystal structure as CH_3_NH_3_PbI_3_. DFT calculations predicted CH_3_NH_3_BaI_3_ to form stable perovskite materials with an estimated band gap of 3.3 eV [118]. In comparison to CH_3_NH_3_PbI_3_ (1.57 eV), the high band gap is caused by the low work function (2.7 eV) and low electronegativity (0.88) of barium [118, 125, 127].

Barium halide perovskites can be synthesized via low-temperature solution- or vapor-based methods [124, 126]; however, the extreme sensitivity towards humidity hampers the synthesis and characterization as well as the applicability in photovoltaics [118].

The structural and optical data of alkaline-earth metal halide perovskites are summarized in Table 5.

**Table 5:** Structural and optical data of alkaline-earth metal halide perovskites

Perovskite	Sim./exp.	Crystal system (space group)	Band gap/eV	References
CH_3_NH_3_MgI_3_	Sim.	Tetragonal	1.5	[49]
CH(NH_2_)_2_MgI_3_	Sim.	Trigonal (*P*3*m*1)	0.9	[49]
CsMgI_3_	Sim.	Orthorhombic	1.7	[49]
CH_3_NH_3_CaI_3_	Sim./exp.	Tetragonal/pseudo-orthorhombic	2.95, 3.78	[47, 118]
CH_3_NH_3_CaI_3−*x*_Cl_*x*_	Exp.	–	–	[47]
CH_3_NH_3_SrI_3_	Sim./exp.	Tetragonal	3.6 eV	[118]
CH_3_NH_3_BaI_3_	Sim.	Tetragonal	3.3 eV	[118]

Dimensionality and PCE values have not been reported for these materials

### Transition metal halide perovskites

Considerable interest in the field of transition metal halide perovskites arises from the rich chemistry and relatively high abundance of transition metals [128]. The multiple oxidation states of transition metals, however, might cause problems with regard to chemical stability [67]. In addition, the small ionic radii of transition metal cations such as Cu^2+^ (73 pm), Fe^2+^ (78 pm), or Pd^2+^ (86 pm) sterically hinder the formation of three-dimensional structures, which leads to lower dimensional layered configurations isostructural to Ruddlesden–Popper perovskites (e.g. K_2_NiF_4_) [51, 128] such as (CH_3_NH_3_)_2_CuCl_*x*_Br_4−*x*_ [53] (CH_3_NH_3_)_2_FeCl_4_ [129], or (CH_3_NH_3_)_2_PdCl_4_ [44].

Transition metal halide perovskites were studied extensively in the last decades, in particular with regard to the magnetic properties [129] and phase transitions [130] resulting from the lower dimensional structures. Various transition metals such as vanadium, manganese, iron, cobalt, nickel, palladium, copper, zinc, cadmium, and mercury have been predicted as promising replacement candidates for lead in the perovskite structure [34, 49, 128]. Various alternative lead-free transition metal halide perovskite materials have been reported [131, 132]. CsNiX_3_ (X = Cl, Br, I), for example, was synthesized via a hydrothermal method to obtain a nickel-based perovskite with a BaNiO_3_ structure consisting of face-sharing NiX_6_ octahedra which are separated by CsX_12_ cuboctahedra [131]. CsNiCl_3_ and CsNiBr_3_, in particular, were predicted to exhibit low electronic band gaps and dispersive band edges making these two compounds attractive for photovoltaics [49]. This hydrothermal synthesis method is also suggested to be extendable to cobalt and iron perovskites [131]. Layered perovskite structures of bis-(alkylammonium) metal(II) tetrahalide (C_*n*_H_2*n*+1_NH_3_)_2_MX_4_ and (*α*,*ω*-)polymethylene diammonium metal(II) tetrahalide NH_3_(CH_2_)_m_NH_3_MX_4_ (M = Cd, Cu, Fe, Mn, Pd and X = Cl, Br) were investigated by Arend et al. [132]. Mercury and cadmium halide perovskites have the same inherent problems of high toxicity as lead-based materials. Despite the toxicity issue of cadmium-based compounds, a hybrid cadmium halide perovskite ((3-pyrrolinium)(CdCl_3_)) with an above-room-temperature ferroelectric behavior and an anomalous photovoltaic effect has been reported recently [133]. The potential of this material for photovoltaic applications is supported by the extraordinary high *V*_OC_ of 32 V of a 1-mm bulky crystal [133]. A more detailed view on transition metal halide perovskites based on copper, iron and palladium is given in the following chapters.

#### Copper halide perovskites

Copper is a non-toxic, low-cost earth abundant transition metal. The divalent Cu^2+^ cation is of particular interest for the incorporation into the perovskite structure as replacement for Pb^2+^ because of its ambient stability and the high absorption coefficient in the visible region [53, 134]. Cu^2+^ has a 3d^9^ 4s^0^ (t_2g_^6^ e_g_^3^) electronic configuration different to the group-14 main group metal cations of Sn^2+^ and Pb^2+^, i.e. lone pair electrons, which has a considerable effect on the electronic band structure [28, 53, 134].

**Fig. 7:**
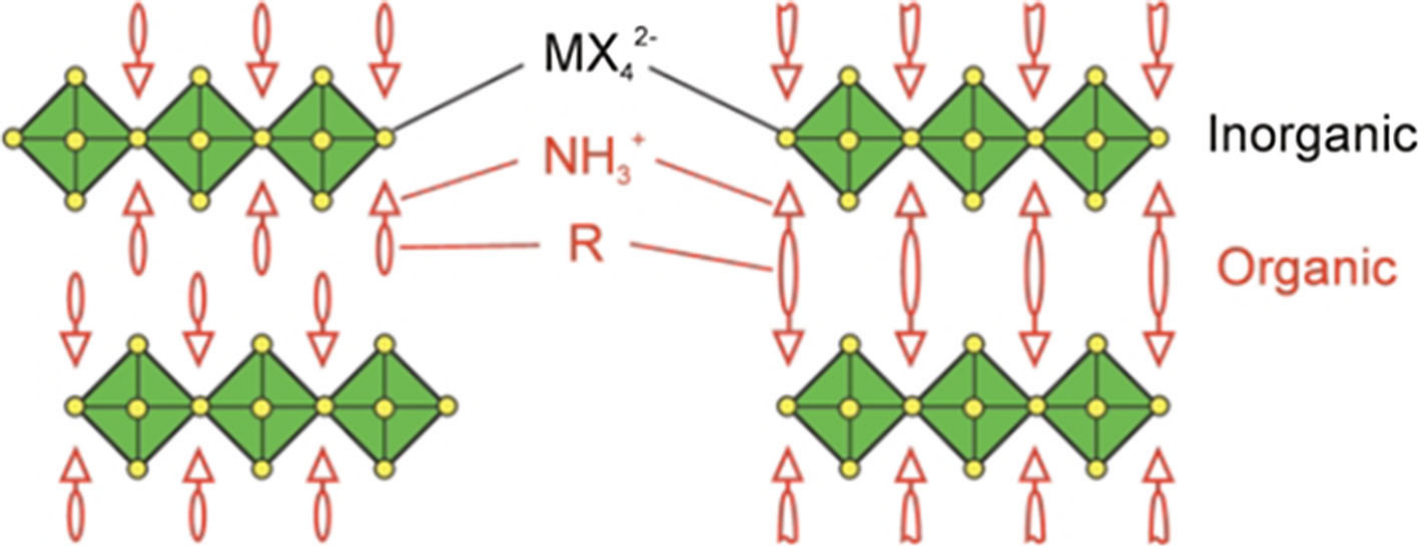
Schematic representation of 〈100〉-oriented perovskites with organic monoammonium ((R-NH_3_)_2_MX_4_,* left*) and diammonium ((NH_3_-R-NH_3_)MX_4_,* right*) cations. Reprinted with permission from [56]. Copyright (2001) Royal Society of Chemistry

Due to the smaller ionic radius of Cu^2+^ (73 pm) compared to Pb^2+^ (119 pm) or Sn^2+^ (110 pm), the formation of three-dimensional structures is sterically hindered, and thus hybrid copper halide perovskites form two-dimensional layered structures, which are isostructural to Ruddlesden–Popper phase compounds [51, 53, 128, 135]. These hybrid perovskites have the general formula (R-NH_3_)_2_CuX_4_ incorporating monovalent ammonium cations (R = alkyl, aryl) and halide counterions [34]. The two-dimensional structures form inorganic layers of corner-sharing BX_6_ octahedra separated by monolayers of organoammonium cations on either side of the metal halide sheets, which are accommodated within the voids of the inorganic framework [34, 135–138]. The layered structure is stabilized by hydrogen bonding interactions (N–H···X) between the ammonium groups and the halogen atoms and by van der Waals interactions between the interdigitating organic moieties [135, 139]. Each successive inorganic perovskite sheet is shifted to give a “staggered” configuration of the layers (Fig. 7, left) [139]. Examples are (CH_3_(CH_2_)_3_NH_3_)_2_CuBr_4_ and (*p*-F-C_6_H_5_C_2_H_4_NH_3_)_2_CuBr_4_ [34].

A further way to stabilize the layered structure is the use of organic diammonium cations (NH_3_^+^-R-NH_3_^+^, R = alkyl, aryl) in (NH_3_-R-NH_3_)CuX_4_ compounds [139]. Diammonium-based layered structures feature hydrogen bonding interactions of both functional ammonium groups to halogen atoms of the inorganic sheets resulting in an “eclipsed” arrangement of the layers, which are separated by a single organic layer instead of a double or bilayer (Fig. 7, right) [139]. The distance between adjacent inorganic layers can be influenced by the length of the organic spacer R, which eventually affects the compound's dimensionality and physical properties [139]. Examples are (ethylenediammonium)CuBr_4_ [137] and [NH_3_(CH_2_)_*n*_NH_3_]CuX (*n* = 2–5, X = Cl_4_, Cl_2_Br_2_) [140].

**Fig. 8:**
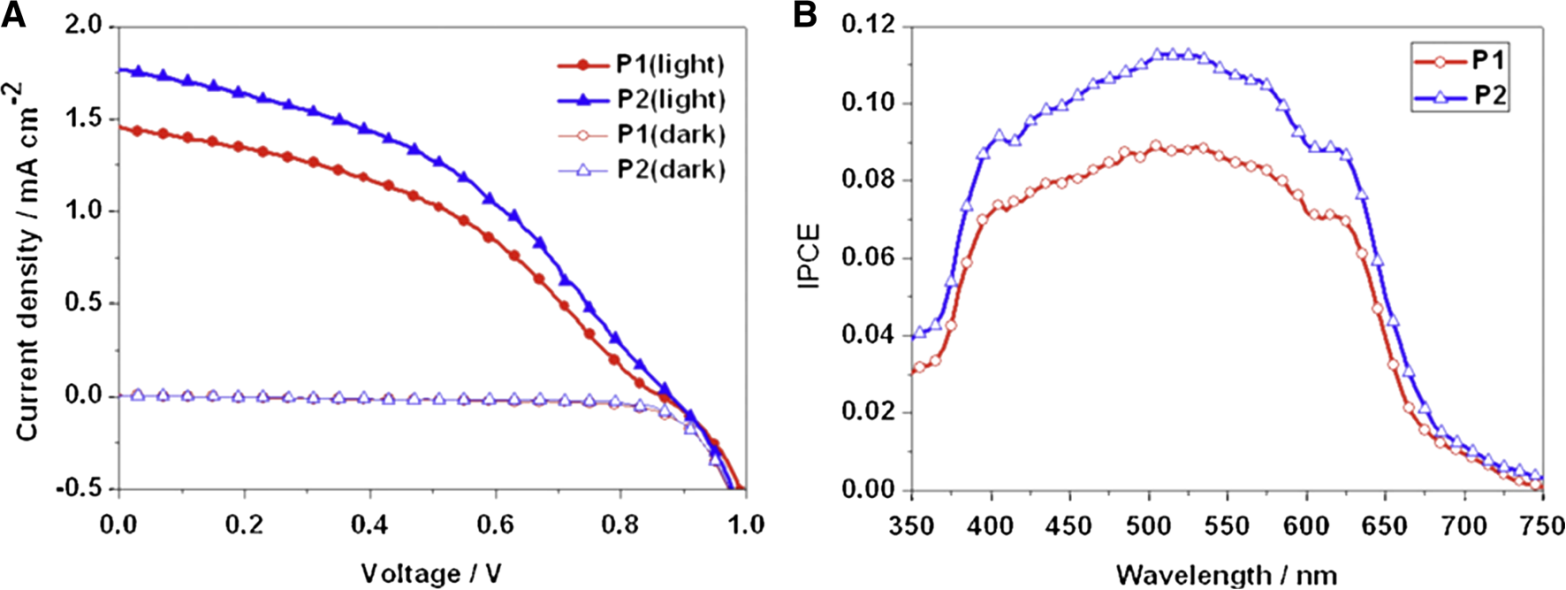
**a** *J*–*V* curves under illuminated and dark conditions and **b** IPCE (incident photon-to-electron conversion efficiency) spectra of copper halide perovskite-based solar cells using (*p*-F-C_6_H_5_C_2_H_4_NH_3_)_2_CuBr_4_ (P1) and (CH_3_(CH_2_)_3_NH_3_)_2_CuBr_4_) (P2) as absorber materials. Adapted with permission from [34]. Copyright (2015) Elsevier

Two-dimensional copper halide perovskites have been investigated with regard to their structural and magnetic properties (e.g. [C_2_H_5_NH_3_]_2_CuCl_4_ [136], 3-ammoniumpyridinium tetrachlorocuprate(II) [137], 3-ammoniumpyridinium tetrabromocuprate(II) [137], bis(morpholinium) tetrachlorocuprate(II) [137]), UV light-induced photochromic behavior (e.g. (C_4_H_9_NH_3_)_2_CuCl_4_ [138]), as intercalation-type cathode material in Li-ion batteries (e.g. (EDBE)[CuCl_4_] with EDBE = 2,2′-(ethylenedioxy) bis(ethylammonium) [141]), and as solution-processable absorber in perovskite solar cells [34, 53]. Cui et al. implemented two-dimensional layered copper perovskites (*p*-F-C_6_H_5_C_2_H_4_NH_3_)_2_CuBr_4_ and (CH_3_(CH_2_)_3_NH_3_)_2_CuBr_4_) as absorber materials in meso-structured perovskite solar cells and obtained PCE values of 0.51 and 0.63%, respectively (Fig. 8) [34]. Both materials were prepared via a low-temperature, solution-based method from CuBr_2_ and the corresponding ammonium bromide compound, i.e. *n*-butylammonium bromide or 4-fluorophenethylammonium bromide, in aqueous hydrobromic acid and exhibited optical band gaps of 1.74 and 1.76 eV, respectively [34].

Cortecchia et al. reported on two-dimensional copper halide perovskites with the general formula (CH_3_NH_3_)_2_CuCl_*x*_Br_4−*x*_ with a varying Br:Cl ratio [53]. Ligand-to-metal charge transfer transitions and Cu d–d transitions influence the absorption properties of this material [128]. In addition, the optical band gap was found to be tunable via the Br:Cl ratio within the visible to near-infrared range with a bathochromic shift for higher bromide content: (CH_3_NH_3_)_2_CuCl_4_ (2.48 eV), (CH_3_NH_3_)_2_CuCl_2_Br_2_ (2.12 eV), (CH_3_NH_3_)_2_CuClBr_3_ (1.90 eV), and (CH_3_NH_3_)_2_CuCl_0.5_Br_3.5_ (1.80 eV) [53]. The as-prepared (CH_3_NH_3_)_2_CuCl_*x*_Br_4−*x*_ perovskites were investigated in solar cells using thick (5 µm) mesoporous TiO_2_ scaffolds giving PCE values of 0.0017% ((CH_3_NH_3_)_2_CuCl_0.5_Br_3.5_) and 0.017% ((CH_3_NH_3_)_2_CuCl_2_Br_2_) [53]. However, the photovoltaic performance of layered copper halide perovskites in general is limited by various factors including low absorption coefficients, the high effective mass of holes and the intrinsic low conductivity of two-dimensional perovskite structures [34, 53].

The choice of the halide counterion plays a key role not only in the engineering of the band gap but is also essential with regard to the material’s stability, film formation properties and photovoltaic performance. Bromide is responsible for the partial reduction of Cu^2+^ to Cu^+^ in the perovskite framework, which is accompanied by the formation of anion vacancies. These crystallographic defects act as electron traps and limit the photovoltaic performance since an additional charge recombination pathway is introduced [128]. This is supported by Cortecchia et al. who found a pronounced photoluminescence with higher bromine contents resulting from the in situ formation of Cu^+^ ions and the corresponding charge carrier recombination at the charge traps [53]. Chloride was found to be essential for the material’s stability against the copper reduction and to improve the crystallization of the perovskite accompanied by a hypsochromic shift of the optical band gap [53].

The presence of the Jahn–Teller active Cu^2+^ metal cation introduces an additional flexibility into the inorganic framework, which also affects hydrogen bonding interactions [28]. This is based on the Jahn–Teller distortion of the CuX_6_ octahedra leading to an elongation of two equatorial Cu–X bonds within the octahedral coordination. As a consequence, the layered perovskite adopts an antiferrodistortive structure in which adjacent Cu^2+^ ions are linked via one short (normal) and one Jahn–Teller elongated (semicoordinate) bond via a bridging halide ion [137]. The normal bond length is relatively constant, whereas the semicoordinate bond is considerably elastic, allowing the inorganic layers to adopt a more flexible structure, which enables the interaction with larger organic ammonium cations to be incorporated into the two-dimensional structure [53, 137]. Other layered perovskite analogues with Jahn–Teller active metal cations such as Cr^2+^ show similar structural distortions and ferromagnetic behavior (e.g. (C_6_H_5_CH_2_NH_3_)_2_CrBr_4_) [142]. The substitution of Cu^2+^ or Cr^2+^ with other divalent metal cations which do not show a Jahn–Teller effect (e.g. Mn^2+^, Fe^2+^, Cd^2+^) causes a rather rigid inorganic framework of the perovskite materials, which exhibit antiferromagnetic behavior [28, 137].

#### Iron halide perovskites

The smaller ionic radius of the divalent Fe^2+^ metal cation (78 pm) compared to Pb^2+^ (119 pm) sterically hinders the formation of three-dimensional structures [51]. Two-dimensional layered structures isostructural to Ruddlesden–Popper perovskites are formed instead [128].

Several two-dimensional iron halide perovskites with a general formula A_2_FeX_4_ have been studied, where A is an organic aliphatic or aromatic ammonium cation and X is a halide counterion [129, 143]. The layered perovskites are made up of alternating stacks of organic (alkyl, aryl) ammonium and inorganic metal–halogen sheets of corner-sharing FeX_6_ octahedra [144]. Even though various hybrid iron halide perovskites such as (RNH_3_)_2_FeCl_4_ (R = CH_3_, C_2_H_5_, C_3_H_7_, C_6_H_5_CH_2_), (CH_3_NH_3_)_2_FeCl_2_Br_2_, (CH_3_NH_3_)_2_FeCl_4_, and (CH_3_NH_3_)_2_FeCl_3_Br have been investigated with regard to their magnetic properties, only a few studies pay attention to the electrical and optical properties [129, 143–145].

Beside limitations of charge transport properties based on two-dimensional structures and inappropriate band gaps for solar cells, a drawback of iron halide perovskites are the multiple oxidation states of iron that limit the material’s stability towards oxidation, i.e. oxidation of Fe^2+^ to Fe^3+^ similar to tin- and germanium-based perovskites [128]. Thus, iron halide perovskites have not been used as absorber material for photovoltaic applications yet.

#### Palladium halide perovskites

Only a few studies on palladium-based perovskites have been reported so far [44, 146, 147]. Most of the investigated palladium halide perovskites exhibit the general formula A_2_PdX_4_, where A is an organic aliphatic or aromatic ammonium cation (RNH_3_^+^) such as CH_3_NH_3_^+^ [44] and *n*-octylammonium [146], and X is a halide [44]. These materials form two-dimensional layered structures consisting of an alternating stack of organic and inorganic layers [44].

Although (CH_3_NH_3_)_2_PdCl_4_ is expected to form a three-dimensional structure according to the Goldschmidt tolerance factor concept (*t* = 0.956, which is clearly within the range for three-dimensional perovskites (0.813–1.107) [45]), Huang et al. found a two-dimensional layered structure [44]. (CH_3_NH_3_)_2_PdCl_4_, which was prepared via a low-temperature solution-based method using CH_3_NH_3_Cl and PdCl_2_ under ambient conditions, exhibits interesting properties for optoelectronic applications with a direct optical band gap of 2.22 eV and shows an absorption coefficient of about 10^4^ cm^−1^ [44]. X-ray diffraction and UV–Vis measurements confirm the improved ambient stability of the material compared to lead- and tin-based perovskites. The authors suggest the substitution of chloride with heavier halides such as bromide or iodide to lower the band gap. Together with the increased oxidation stability and promising optical properties, this could be a promising example of a palladium halide perovskites for optoelectronic applications.

Cheng et al. synthesized (C_8_H_17_NH_3_)_2_PdCl_4_ using *n*-octylammonium chloride and PdCl_2_, which exhibits a similar two-dimensional layered structure as the methyl-ammonium analogue [146]. The inorganic layers consist of a PdCl_4_^2−^ network and are sandwiched by organic *n*-octylammonium cations. This perovskite material was used as template for preparing self-assembled, ultrathin palladium nanosheets [146].

In addition, rigid layered structures with high crystallinity can be prepared using PdCl_2_ and propylammonium-functionalized silsesquioxane under ambient conditions. The hybrid palladium halide perovskite material exhibits two-dimensional structures consisting of corner-sharing PdCl_4_^2−^ octahedra and organic interlayers of alkylammonium functional silsesquioxane with a cage-like structure [147]. The material showed excitonic absorption/emission properties similar to other layered lead-based perovskites (PbCl_4_^2−^). In addition, the silsesquioxane produces a microporous scaffold between the inorganic metal halide layers that can be filled with molecules. Similar approaches are reported for copper, lead, and manganese forming hybrid silsesquioxane–metal halide perovskites with porous structures [147].

Table 6 gives an overview about structural and optical data of transition metal and europium halide perovskites and their performance as absorber material in solar cells.

**Table 6:** Structural and optical data of transition metal and europium halide perovskites and the obtained PCEs (if applied in photovoltaic devices)

Perovskite	Sim./exp.	Crystal system (space group)	Dimensionality	Band gap/eV	PCE/%	References
(*p*-F-C_6_H_5_C_2_H_4_NH_3_)_2_CuBr_4_	Exp.	–	2D	1.74	0.51	[34]
(CH_3_(CH_2_)_3_NH_3_)_2_CuBr_4_	Exp.	–	2D	1.76	0.63	[34]
(CH_3_NH_3_)_2_CuCl_4_	Exp.	Monoclinic (*P*12_1_/*a*1)	2D	2.48	–	[53]
(CH_3_NH_3_)_2_CuCl_2_Br_2_	Exp.	Orthorhombic (*Acam*)	2D	2.12	0.017	[53]
(CH_3_NH_3_)_2_CuClBr_3_	Exp.	Orthorhombic (*Acam*)	2D	1.90	–	[53]
(CH_3_NH_3_)_2_CuCl_0.5_Br_3.5_	Exp.	Orthorhombic (*Acam*)	2D	1.80	0.0017	[53]
(CH_3_NH_3_)_2_FeCl_4_	Exp.	Orthorhombic (*Pccn*) <335 K	2D	–	–	[129, 143, 144]
		Tetragonal (*I*4/*mmm*) >335 K	**–**			
(C_2_H_5_NH_3_)_2_FeCl_4_	Exp.	–	2D	–	–	[129]
(C_3_H_7_NH_3_)_2_FeCl_4_	Exp.	–	2D	–	–	[129]
(C_6_H_5_CH_2_NH_3_)_2_FeCl_4_	Exp.	–	2D	–	–	[129]
(CH_3_NH_3_)_2_FeCl_2_Br_2_	Exp.	–	2D	–	–	[143]
(CH_3_NH_3_)_2_FeCl_3_Br	Exp.	–	2D	–	–	[145]
(CH_3_NH_3_)_2_PdCl_4_	Exp.	–	2D	2.22	–	[44]
(C_8_H_17_NH_3_)_2_PdCl_4_	Exp.	–	2D	–	–	[146]
CH_3_NH_3_EuI_3_	Exp.	–	3D	–	–	[148]
(C_4_H_9_NH_3_)_2_EuI_4_	Exp.	–	2D	–	–	[148, 149]

### Lanthanide and actinide halide perovskites

Rare earth metal ions have been used as substituent for Pb^2+^ giving rise towards lanthanide and actinide halide perovskites [148, 149]. Liang and Mitzi investigated a novel class of luminescent europium halide perovskites: CH_3_NH_3_EuI_3_ is a three-dimensional ABX_3_-type perovskite with a tetragonally distorted structure of BX_6_ corner-connected octahedra, which can be synthesized via a diffusion-based solid-state synthesis route from CH_3_NH_3_I and EuI_2_ [63]. (C_4_H_9_NH_3_)_2_EuI_4_ is a two-dimensional A_2_BX_4_-type perovskite adopting a layered structure of corner-sharing BX_6_ octahedra sandwiched by organic butylammonium cations on both sides of the metal halide sheets [149]. The material was made by a low-temperature (ca. 140–160 °C) solid-state reaction of C_4_H_9_NH_2_·HI and EuI_2_ [148]. Solution-based synthesis routes are limited by the oxidation instability of Eu^2+^, and by the strong tendency of Eu^2+^ to bind solvent molecules, thereby impeding the perovskite formation. However, both structure types are characterized by a sixfold Eu(II) coordination, i.e. EuI_6_ octahedra. The authors expect both families of compounds to be interesting materials for hybrid optoelectronic devices such as light-emitting diodes [148] (see also Table 6).

In addition, rare earth metal ions are commonly used as dopants in ABX_3_-type perovskites. In particular, alkaline-earth metal halide perovskites of the family CsBX_3_ (B = Mg, Ca, Sr; X = Cl, Br, I) have been investigated with regard to their optical properties (e.g. photoluminescence) due to doping with rare earth metal ions such as Eu^2+^ [119–121], Tm^2+^ [122], and Yb^2+^ [123]. In case of CsBI_3_:Eu^2+^ and CsBBr_3_:Eu^2+^ (B = Mg, Ca, Sr), divalent Eu^2+^ metal cations occupy the sixfold, octahedrally coordinated alkaline-earth metal sites of the host compound [120, 121]. For thulium- and ytterbium-doped perovskites, the situation is quite similar [122, 123]. The applicability of these luminescent materials, for example in optoelectronic devices, is, however, limited because of the sensitivity towards moisture [121]. Nevertheless, lanthanide-based perovskites are expected to have interesting optical properties and, therefore, might be potential candidates for novel absorber materials for photovoltaics [50].

In addition, lanthanides (e.g. La^3+^, Ce^3+^, Pr^3+^, Nd^3+^, Sm^3+^, Eu^3+^, Gd^3+^, Dy^3+^, Er^3+^, Tm^3+^, Lu^3+^) and actinides (e.g. Pu^3+^, Am^3+^, Bk^3+^) have been employed in quaternary halide double perovskites [63, 150], but till now no studies on their photovoltaic properties have been reported.

## Heterovalent substitution with mono-, tri- and tetravalent cations

Heterovalent substitution is a second viable approach towards alternative lead-free perovskite materials. It is based on the replacement of the divalent lead cation with a cation in a different valence state, e.g. a mono-, tri- or tetravalent cation. Due to the different valence state, no straightforward substitution with heterovalent cations is possible. Therefore, two different procedures for heterovalent replacement can be distinguished: The first method, the mixed-valence approach, is based on a mixture of an equal number of mono- and trivalent cations to give an average overall valence state of +2 as present in Pb^2+^. Examples for perovskites following the mixed-valence approach are thallium [58, 59] and gold [60–62] halide perovskites. The second method is based on the heterovalent substitution of the divalent Pb^2+^ with trivalent cations such as Sb^3+^ and Bi^3+^ [35, 36, 55, 65, 66]. However, this is accompanied with a considerable change in the structure from ABX_3_-type to A_3_B_2_X_9_-type perovskites to maintain charge neutrality.

Enormous progress in the development of novel lead-free perovskite semiconductors might arise from the heterovalent substitution approach since further non-divalent cations become amenable. In the next section, we give a general view on the structural diversity of heterovalently substituted metal halide perovskites ranging from zero-dimensional to three-dimensional systems, highlight remarkably interesting optoelectronic properties and discuss the recent progress in the field of photovoltaic applications of this class of semiconductors.

### Thallium halide perovskites

Thallium is a p-block metal with a Tl^+^ cation isoelectronic to Pb^2+^ (6s^2^ 6p^0^ electronic configuration). The monovalent Tl^+^ cation, however, cannot substitute the divalent Pb^2+^ metal cation directly in ABX_3_-type perovskites because of the violation of the charge neutrality between cationic and anionic species. Nevertheless, the incorporation of thallium into the perovskite structure is possible via the mixed-valence approach using Tl^+^ (6s^2^) and Tl^3+^ (6s^0^) [28]. An example for such a mixed-valent thallium halide perovskite is ${\text{CsTl}}_{0.5}^{ + } {\text{Tl}}_{0.5}^{3 + } {\text{X}}_{ 3}$ (X = F, Cl), where the mono- and trivalent thallium cations are accommodated in a charge-ordered perovskite structure [58]. This class of thallium halide perovskites was investigated in terms of superconductive behavior by Retuerto et al. and Yin et al. [58, 59]. With regard to the optical properties, the optical band gap of CsTlCl_3_ was experimentally determined to be approximately 2.5 eV [58].

A further interpretation of the mixed-valence approach involves the incorporation of two different metal cations in a different valence state. An example thereof is the mixed thallium–bismuth halide perovskite CH_3_NH_3_Tl_0.5_Bi_0.5_I_3_, where Pb^2+^ metal cation units of the lead-based analogue CH_3_NH_3_PbI_3_ are replaced with Tl^+^/Bi^3+^ heterovalent ionic pairs [151]. Giorgi et al. theoretically investigated this lead-free hybrid perovskite with regard to its structural and electronic properties via DFT analysis and calculated a direct band gap of 1.68 eV [151]. According to these calculations, CH_3_NH_3_Tl_0.5_Bi_0.5_I_3_ is predicted to be a potential alternative solar cell material. However, despite these quite promising considerations and optical properties (Table 7), thallium-based compounds are presumably no alternative to lead-based perovskites in terms of photovoltaic applications due to the inherent toxicity of thallium.

**Table 7:** Structural and optical data of thallium and gold halide perovskites

Perovskite	Sim./exp.	Crystal system (space group)	Dimensionality	Band gap/eV	References
CsTlF_3_ $({\text{CsTl}}_{0.5}^{ + } {\text{Tl}}_{0.5}^{3 + } {\text{F}}_{ 3} )$	Exp.	Cubic ($Fm\bar{3}$ *m*)	3D	–	[58, 59]
CsTlCl_3_ $({\text{CsTl}}_{0.5}^{ + } {\text{Tl}}_{0.5}^{3 + } {\text{F}}_{ 3} {\text{Cl}}_{ 3} )$	Exp.	Tetragonal (*I*4/*m*) Cubic (*Fm* $\bar{3}$ *m*)	3D	ca. 2.5	[58, 59]
CH_3_NH_3_Tl_0.5_Bi_0.5_I_3_	Sim.	Tetragonal	–	1.68	[151]
Cs_2_Au^I^Au^III^Cl_6_	Exp.	Tetragonal (*I*4/*mmm*)	3D	2.04	[60, 152]
Cs_2_Au^I^Au^III^Br_6_	Exp.	Tetragonal (*I*4/*mmm*)	3D	1.60	[152, 153]
Cs_2_Au^I^Au^III^I_6_	Exp.	Tetragonal (*I*4/*mmm*)	3D	1.31	[61, 152, 153]
[NH_3_(CH_2_)_7_NH_3_]_2_[(Au^I^I_2_)(Au^III^I_4_)(I_3_)_2_]	Exp.	Triclinic (*P* $\bar{1}$)	2D	0.95	[62]
[NH_3_(CH_2_)_8_NH_3_]_2_[(Au^I^I_2_)(Au^III^I_4_)(I_3_)_2_]	Exp.	Monoclinic (*C*2/*m*)	2D	1.14	[62]

These materials have not been implemented in photovoltaic devices so far

### Gold halide perovskites

Gold halide perovskites are similar to thallium-based analogues amenable via the mixed-valence approach. Consequently, gold has to be present in a mixture of monovalent Au^+^ (5d^10^, t_2g_^6^ e_g_^4^) and trivalent Au^3+^ (5d^8^, t_2g_^6^ e_g_^2^) metal cations to form ABX_3_-type perovskite structures [28], like in the case of Cs_2_Au^I^Au^III^X_6_ (X = Cl, Br, I) compounds [60, 61, 152, 153]. Additionally, hybrid gold halide perovskites have been investigated such as [NH_3_(CH_2_)_7_NH_3_]_2_[(Au^I^I_2_)(Au^III^I_4_)(I_3_)_2_] and [NH_3_(CH_2_)_8_NH_3_]_2_[(Au^I^I_2_)(Au^III^I_4_)(I_3_)_2_] [62].

Due to the presence of mono- and trivalent metal cations, two different coordination spheres are present in mixed-valent gold halide perovskites, i.e. linear (twofold) and square-planar (fourfold) coordination of Au^+^ and Au^3+^, respectively. In the case of Cs_2_Au^I^Au^III^X_6_ (X = Cl, Br, I), the crystal structure is derived from a distorted ABX_3_-type perovskite consisting of BX_2_ (linear [Au^I^X_2_]^−^ unit) and BX_4_ (square-planar [Au^III^X_4_]^−^ unit) building blocks [60, 61, 152–154]. The BX_2_ and BX_4_ units arrange alternately to accomplish the nominal octahedral coordination in the perovskite structure. While linearly coordinated [Au^I^X_2_]^−^ units are completed by neighboring [Au^III^X_4_]^−^ units via four coplanar halide ions forming compressed octahedra, square-planar coordinated [Au^III^X_4_]^−^ units are completed by apical [Au^I^X_2_]^−^ units via two halide ions forming elongated octahedra. The resulting distorted three-dimensional perovskite network can, therefore, be expressed as Cs_2_[Au^I^X_2_][Au^III^X_4_] [61, 62, 153].

The hybrid mixed-valent gold halide perovskites [NH_3_-R-NH_3_]_2_[(Au^I^I_2_)(Au^III^I_4_)(I_3_)_2_] (R = heptyl, octyl) feature inorganic metal halide sheets of corner-sharing octahedra which are separated by organic diammonium cations (e.g. (NH_3_(CH_2_)_7_NH_3_)^2+^) and (NH_3_(CH_2_)_8_NH_3_)^2+^) to give a layered two-dimensional structure. The nominal octahedral coordination of the Au^I^ center within the [Au^I^I_2_]^−^ units is accomplished by neighboring [Au^III^I_4_]^−^ units via four coplanar halide ions forming compressed octahedra, while [Au^III^I_4_]^−^ units are completed by two asymmetric triiodide ions (I_3_^−^) in apical position forming elongated nominal octahedra [62]. The distorted nominal AuI_6_ octahedra are corner connected to give a layered structure separated by organic interlayers.

Mixed-valent gold halide perovskites such as Cs_2_Au^I^Au^III^X_6_ were predominantly investigated in terms of superconductivity [61]. Further research studies mainly focus on the structural characterization as well as on the electronic and optical behavior [60–62, 152, 153]. With regard to the optical properties, the choice of the halide counterion plays an essential role for band gap engineering in mixed-valent systems such as Cs_2_Au^I^Au^III^X_6_ (X = Cl, Br, I). By substitution of chlorine with bromine or iodine, the optical band gap can be shifted to lower values. Liu et al. determined the optical band gaps of the corresponding perovskites via optical reflectivity measurements to be 2.04 eV (X = Cl), 1.60 eV (X = Br), and 1.31 eV (X = I) [152]. Cs_2_Au^I^Au^III^I_6_, in particular, is a promising absorber material for photovoltaic applications due to the almost ideal band gap according to the Shockley–Queisser limit, and the three-dimensional distorted ABX_3_-type perovskite structure similar to lead-based analogues [155]. To the best of our knowledge, however, this class of materials was not characterized with regard to its photovoltaic performance so far.

Castro-Castro et al. investigated the optical properties of two-dimensional layered hybrid gold halide perovskites including [NH_3_(CH_2_)_7_NH_3_]_2_[(Au^I^I_2_)(Au^III^I_4_)(I_3_)_2_] and [NH_3_(CH_2_)_8_NH_3_]_2_[(Au^I^I_2_)(Au^III^I_4_)(I_3_)_2_], and determined band gaps of 0.95 and 1.14 eV, respectively [62], which are lower than in the three-dimensional Cs_2_Au^I^Au^III^I_6_ perovskite (1.31 eV). These unusual low band gaps—lower dimensional perovskites usually exhibit higher band gaps [28]—can be explained by additionally induced electronic interactions between the [Au^I^I_2_]^−^ and [Au^III^I_4_]^−^ units and I_3_^−^ ions, which are absent in Cs_2_Au^I^Au^III^I_6_ [62].

The properties of the aforementioned perovskites are summarized in Table 7. Further examples of mixed-valent perovskite materials employing gold together with the pnictogens antimony and bismuth in double perovskite structures are given below.

### Antimony halide perovskites

Antimony halide perovskites are a potential alternative to lead-based perovskite semiconductors for photovoltaic applications to address the chemical stability and the toxicity issue [55]. The trivalent Sb^3+^ metal cation (1) is isoelectronic to Sn^2+^ (4d^10^ 5s^2^) and has a similar s^2^ valence electronic configuration as Pb^2+^ (5s^2^ lone pair), (2) has a comparable electronegativity (Sb: 2.05, Sn: 1.96, Pb: 2.33) but (3) a significant smaller ionic radius (76 pm) compared to the divalent Sn^2+^ (110 pm) and Pb^2+^ (119 pm) metal cations [35, 51, 125, 156].

**Fig. 9:**
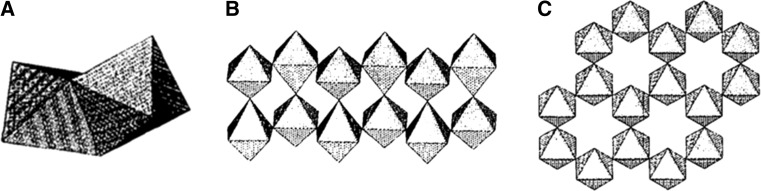
Anionic sublattices present in antimony halide perovskites in polyhedral representation: **a** zero-dimensional dimers of face-sharing octahedra, **b** one-dimensional double chains of corner-connected octahedra, and **c** two-dimensional double-layered structures of corner-sharing octahedra. Reproduced with permission of the International Union of Crystallography [159]. Copyright (1996) International Union of Crystallography

Because of the difference in the oxidation state, antimony halide perovskites have the basic formula A_3_Sb_2_X_9_ (X = Cl, Br, I), where A are organic (e.g. NH_4_^+^ [157], CH_3_NH_3_^+^ [55, 158], dimethylammonium [159], trimethylammonium [160], tetramethylammonium [158], guanidinium [161]) or inorganic (e.g. Rb^+^ [35, 162], Cs^+^ [65, 162–164]) cations. The structural chemistry and dimensionality of antimony halide perovskites are significantly influenced by the choice of cationic and anionic species. Depending on the dimensionality, the crystal structures of antimony-based perovskites featuring Sb_2_X_9_^3−^ enneahalide ions within the anionic sublattice can be divided into three categories (Fig. 9) [159, 163]:

1. zero-dimensional, isolated double octahedral structures comprising pairs of face-sharing SbX_6_ octahedra, which form discrete complex anionic metal halide Sb_2_X_9_^3−^ clusters arranged in dimer units (e.g. (CH_3_NH_3_)_3_Sb_2_I_9_ [55], [N(CH_3_)_4_]_3_Sb_2_Cl_9_ [158], Cs_3_Sb_2_I_9_ [163–165]);
2. infinite one-dimensional double chains of corner-sharing SbX_6_ octahedra forming zigzag-type polyanionic Sb_2_X_9_^3−^ sublattices (e.g. (CH_3_NH_3_)_3_Sb_2_Cl_9_ [166]);
3. two-dimensional corrugated double-layered polyanionic structures based on corner-connected SbX_6_ octahedra to give Sb_2_X_9_^3−^ sub-units (e.g. (NH_4_)_3_Sb_2_I_9_ [157], [NH(CH_3_)_3_]_3_Sb_2_Cl_9_ [160], Rb_3_Sb_2_I_9_ [35], Cs_3_Sb_2_I_9_ [163–165]).

In addition, the processing methodology has an influence on the obtained structure. For example, in the case of Cs_3_Sb_2_I_9_, zero-dimensional dimer species are obtained from solution-based methods, while two-dimensional layered perovskites can be prepared by co-evaporation or solid-state reactions [65]. Due to the prevalence of polymorphism (e.g. [NH_2_(CH_3_)_2_]_3_Sb_2_Cl_9_ [159], Rb_3_Sb_2_I_9_ [35, 162], Cs_3_Sb_2_I_9_ [163]) in this class of perovskites, this dependence of the dimensionality on the processing parameters is an important issue to improve the materials properties (e.g. charge transport) for photovoltaic applications.

A variety of antimony halide perovskites has been investigated with regard to the crystal structure [157, 160, 164, 166], phase transitions of polymorphous compounds [158, 159, 163, 165, 166], as well as ferroelectric and optical properties [162, 167, 168]. Only a few studies aim at a photovoltaic application [35, 55, 65].

**Fig. 10:**
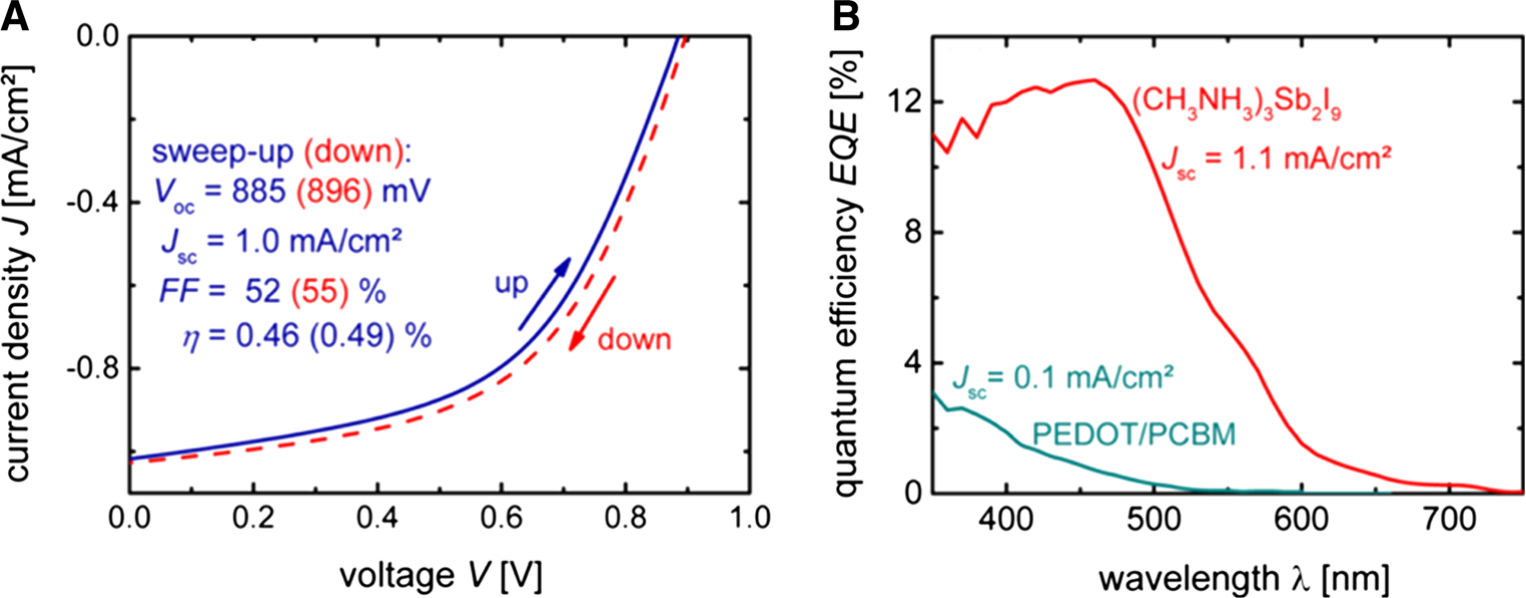
**a** *J*–*V* curves of (CH_3_NH_3_)_3_Sb_2_I_9_-based perovskite solar cells scanned in forward and reverse direction, and **b** corresponding EQE spectra including a reference device without absorber material. Adapted with permission from [55]. Copyright (2016) American Chemical Society

The optoelectronic properties of (CH_3_NH_3_)_3_Sb_2_I_9_ have been investigated by Hebig et al. recently [55]. The compound has a zero-dimensional dimer structure comprising discrete bi-octahedral metal halide units Sb_2_I_9_^3−^ of face-sharing BI_6_ octahedra surrounded by CH_3_NH_3_^+^ cations to balance the charge neutrality. The complex anionic clusters are interconnected via hydrogen bonding interactions of the type N–H···I. (CH_3_NH_3_)_3_Sb_2_I_9_ was prepared via a solution-based deposition method from CH_3_NH_3_I and SbI_3_ at low temperatures (100–120 °C). The peak absorption coefficient is approximately 10^5^ cm^−1^ and thereby in a similar range compared to the lead-based analogue [169]. The optical band gap was determined to be 2.14 eV assuming a direct band transition. (CH_3_NH_3_)_3_Sb_2_I_9_ was implemented as absorber material in planar heterojunction solar cells (ITO/PEDOT:PSS/(CH_3_NH_3_)_2_Sb_2_I_9_ (300 nm)/PC_61_BM/ZnO-NP/Al) to yield a *V*_OC_ of 890 mV, a *J*_SC_ of 1.1 mA cm^−2^, a FF of 55%, and a PCE of ca. 0.5% (Fig. 10). In addition, a maximum external quantum efficiency (EQE) of about 12%, and only little hysteresis in planar perovskite solar cells are reported [55]. The authors attributed this low photocurrent density to an inefficient charge extraction, which might be improved using mesoporous scaffolds.

Peresh et al. investigated the optical properties of inorganic A_3_Sb_2_Br_9_-type antimony halide perovskites and determined band gaps of 2.48 eV (A = Rb^+^) and 2.30 eV (A = Cs^+^) [162]. By substitution of Br^−^ with the heavier I^–^, the band gap can be shifted down to 1.89 eV for Cs_3_Sb_2_I_9_, which is a promising value for photovoltaic applications.

Saparov et al. examined Cs_3_Sb_2_I_9_ as prospective absorber material in solar cells and found improved stability properties under ambient conditions compared to lead and tin halide perovskite films [65]. Cs_3_Sb_2_I_9_ exists in two polymorphs: (1) a zero-dimensional dimer modification (hexagonal) featuring Sb_2_I_9_^3−^ bi-octahedral units and (2) a two-dimensional layered modification (trigonal) [163]. The dimer can be synthesized via solution-based methods using polar solvents, while the layered modification is obtained through solid-state reactions, gas phase reactions (e.g. co-evaporation or sequential deposition of CsI and SbI_3_, followed by annealing in SbI_3_ vapor) or solution-based methods (e.g. crystallization from methanol or non-polar solvents) [65, 163]. According to electronic band structure calculations, the dimer modification has an indirect band gap of 2.40 eV (HSE, Heyd–Scuseria–Ernzerhof), while the layered polymorph exhibits a nearly direct band gap of 2.06 eV (HSE). This latter value is in good agreement with the experimental value of 2.05 eV found for the layered polymorph [65]. Saparov et al. investigated the layered modification of Cs_3_Sb_2_I_9_ as light absorber in perovskite solar cells with the general device architecture of FTO/c-TiO_2_/Cs_3_Sb_2_I_9_/PTAA/Au (PTAA: poly[bis(4-phenyl)(2,4,6-trimethylphenyl)amine]). The material exhibited a rather poor photovoltaic performance with a *V*_OC_ up to 300 mV, a *J*_SC_ below 0.1 mA cm^−2^ and a low overall performance (<1%) [65].

**Fig. 11:**
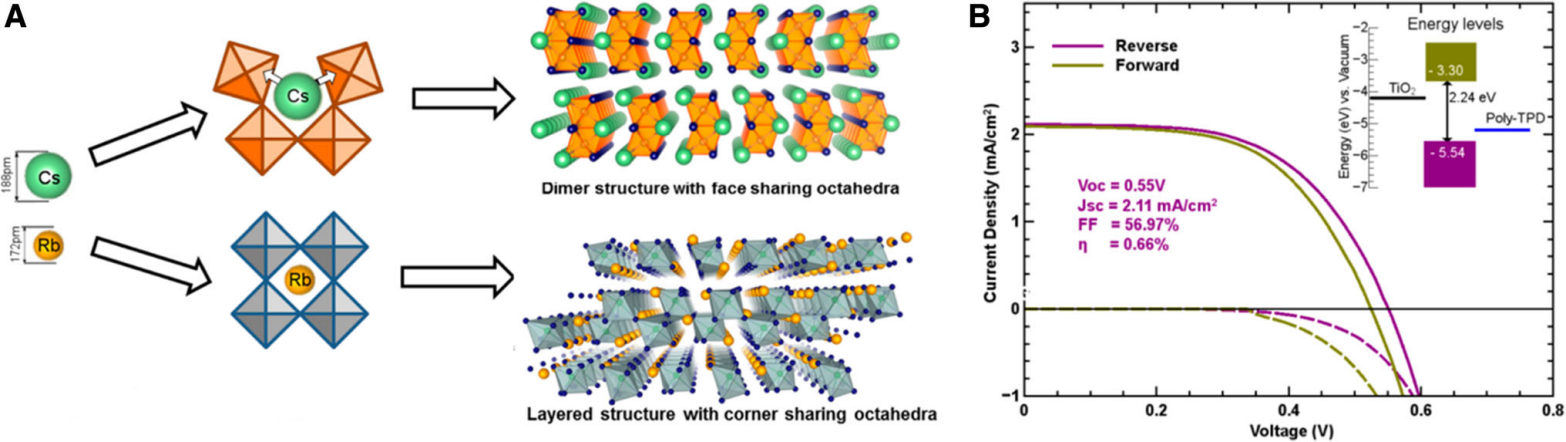
**a** Schematic representation of the influence of the ionic radius of the A-site cation on the structure and dimensionality of A_3_Sb_2_I_9_-type perovskite compounds, and **b** *J*–*V* curves of Rb_3_Sb_2_I_9_-based solar cells under illuminated and dark conditions in *forward* and *reverse* scan direction (*inset* energy level diagram). Reprinted with permission from [35]. Copyright (2016) American Chemical Society

Harikesh et al. have recently reported the synthesis of Rb_3_Sb_2_I_9_ in a layered perovskite structure via a low-temperature solution-based route through the reaction of RbI and SbI_3_ [35]. In comparison to the dimer modification of Cs_3_Sb_2_I_9_, the substitution of Cs^+^ (188 pm) with the smaller Rb^+^ (172 pm) cation was shown to effectively stabilize the structure in the layered modification. As a consequence, the respective Rb_3_Sb_2_I_9_ perovskite forms a two-dimensional layered structure consisting of corner-sharing BX_6_ octahedra, which is different to the zero-dimensional dimer modification of Cs_3_Sb_2_I_9_ comprising isolated bi-octahedral metal halide units B_2_X_9_^3−^ (Fig. 11) [35, 51].

The substitution of Cs^+^ with Rb^+^ in A_3_Sb_2_X_9_-type perovskites is accompanied by only a small blueshift of the band gap. Experimentally, an indirect band gap of 2.1 eV and a direct transition at 2.24 eV was determined for Rb_3_Sb_2_I_9_ [35] compared to a value of 2.05 eV for the band gap of the cesium analogue [65]. In addition, Rb_3_Sb_2_I_9_ exhibits an absorption coefficient over 1 × 10^5^ cm^−1^, which is in a similar range compared to lead-based systems [169]. Harikesh et al. examined solution-processed Rb_3_Sb_2_I_9_ perovskite absorbers in solar cells with an FTO/c-TiO_2_/mp-TiO_2_/Rb_3_Sb_2_I_9_/poly-TPD/Au device architecture (poly-TPD: poly[*N*,*N*′-bis(4-butylphenyl)-*N*,*N*′-bisphenylbenzidine]). The solar cells exhibited a *V*_OC_ of 0.55 V, a *J*_SC_ of 2.12 mA cm^−2^, and a FF of 57% resulting in a PCE of 0.66% (Fig. 11) [35]. These are quite promising results for alternative lead-free perovskite semiconductors.

Mitzi et al. investigated metal-deficient antimony and bismuth-based hybrid perovskites with the chemical formula (H_2_AEQT)B_2/3_I_4_ (B = Sb, Bi; AEQT = 5,5′′′-bis-(aminoethyl)-2,2′:5′,2′′:5′′,2′′′-quaterthiophene) [57]. This class of layered perovskites consists of inorganic metal-deficient metal halide layers (B_2/3_X_4_^2−^) alternating with layers of the organic H_2_AEQT^2+^ cation to form a two-dimensional structure [57]. In addition, vacancies on the metal site within the inorganic sheets together with the rigid organic AEQT-based layers were found to play an essential role in stabilizing the two-dimensional metal-deficient perovskite structure [57].

Antimony halide double perovskite semiconductors with a basic formula A_2_B^I^B^II^X_6_ have been investigated in a theoretical study by Volonakis et al. [63]. These materials are based on a heterovalent substitution of Pb^2+^ with an equal number of mono- and trivalent cations to maintain the charge neutrality and form double perovskite structures (elpasolite structure). Volonakis et al. examined halide double perovskites based on monovalent noble metals (B^I^ = Cu^+^, Ag^+^, Au^+^) and trivalent pnictogen cations (B^II^ = Sb^3+^, Bi^3+^) with Cs^+^ as A-site cation and halide (X = Cl, Br, I) as counterions [63]. The noble-metal and pnictogen cations occupy the B^I^ and B^II^ sites, which alternate along the three crystallographic axes giving rock-salt ordering [63]. The calculated electronic band gaps of the examined antimony halide double perovskites are indirect band gaps and tunable in the visible range, i.e. 0.9–2.1 eV (Cs_2_CuSbX_6_), 1.1–2.6 eV (Cs_2_AgSbX_6_), and 0.0–1.3 eV (Cs_2_AuSbX_6_) [63].

A summary of structural and optical data of antimony halide perovskites and their performance as absorber material in solar cells is given in Table 8.

**Table 8:** Structural and optical data of antimony halide perovskites and the obtained PCEs (if applied in photovoltaic devices)

Perovskite	Sim./exp.	Crystal system (space group)	Dimensionality	Band gap/eV	PCE/%	References
(NH_4_)_3_Sb_2_I_9_	Exp.	Monoclinic (*P*2_1_/*n*)	2D	–	–	[157]
(CH_3_NH_3_)_3_Sb_2_Cl_9_	Exp.	Orthorhombic (*Pmcn*)	1D	–	–	[158, 166]
(CH_3_NH_3_)_3_Sb_2_Br_9_	Exp.	Trigonal (*P* $\bar{3}$ *m*1)	2D	–	–	[158]
(CH_3_NH_3_)_3_Sb_2_I_9_	Exp.	Hexagonal (*P*6_3_/*mmc*)	0D	2.14	ca. 0.5	[55, 163]
[NH_2_(CH_3_)_2_]_3_Sb_2_Cl_9_	Exp.	Monoclinic (*Pc*) at 200 K	–	–	–	[159]
		Monoclinic (*P*2_1_/*c*) at 298 K	2D	–	–	[159, 168]
[NH_2_(CH_3_)_2_]_3_Sb_2_Br_9_	Exp.	Monoclinic (*P*2_1_/*c*)	–	–	–	[167]
[NH(CH_3_)_3_]_3_Sb_2_Cl_9_	Exp.	Monoclinic (*Pc*)	2D	–	–	[160]
[N(CH_3_)_4_]_3_Sb_2_Cl_9_	Exp.	Hexagonal (*P*6_3_/*mmc*)	0D	–	–	[158]
[N(CH_3_)_4_]_3_Sb_2_Br_9_	Exp.	Hexagonal (*P*6_3_/*mmc*)	0D	–	–	[170]
(C_5_H_5_NH)_3_Sb_2_Cl_9_	Exp.	Monoclinic (*C*2/*c*)	1D	–	–	[170]
Rb_3_Sb_2_Br_9_	Exp.	Trigonal (*P* $\bar{3}$ *m*1)	–	2.48	–	[162]
Rb_3_Sb_2_I_9_	Sim./exp.	Monoclinic (*Pc*)	2D	2.1	0.66	[35]
		Monoclinic (*Pc*)	–	1.94	–	[162]
α-Cs_3_Sb_2_Cl_9_	Exp.	Trigonal (*P*321)	2D	–	–	[171]
β-Cs_3_Sb_2_Cl_9_	Exp.	Orthorhombic (*Pmcn*)	1D	–	–	[172]
Cs_3_Sb_2_Br_9_	Exp.	Trigonal (*P* $\bar{3}$ *m*1)	–	2.30	–	[162]
Cs_3_Sb_2_I_9_	Sim./exp.	Hexagonal (*P*6_3_/*mmc*)	0D	1.89–2.4	<1	[65, 162–164]
		Trigonal (*P* $\bar{3}$ *m*1)	2D	2.05		[65]
Cs_2_CuSbX_6_ (X = Cl, Br, I)	Sim.	Cubic (*Fm* $\bar{3}$ *m*)	3D	2.1 (X = Cl)	–	[63]
				1.6 (X = Br)		
				0.9 (X = I)		
Cs_2_AgSbX_6_ (X = Cl, Br, I)	Sim.	Cubic (*Fm* $\bar{3}$ *m*)	3D	2.6 (X = Cl)	–	[63]
				1.9 (X = Br)		
				1.1 (X = I)		
Cs_2_AuSbX_6_ (X = Cl, Br, I)	Sim.	Cubic (*Fm* $\bar{3}$ *m*)	3D	1.3 (X = Cl)	–	[63]
				0.7 (X = Br)		
				0 (X = I)		
(H_2_AEQT)Sb_2/3_I_4_	Exp.	Monoclinic (*C*2/*m*)	2 D	–	–	[173]
[C(NH_2_)_3_]_3_[Sb_2_I_9_]	Exp.	Orthorhombic (*Cmcm*) at 293 K	–	–	–	[161]
	Exp.	Orthorhombic (*Cmcm*) at 348 K				[161]
	Exp.	Hexagonal (*P*6_3_/*mmc*) at 364 K				[161]

### Bismuth halide perovskites

The group-15 metal bismuth is an interesting replacement candidate for lead and tin, which is supported by various aspects [174]: The trivalent Bi^3+^ ion (1) is isoelectronic to Pb^2+^ (6s^2^ 6p^0^ electronic configuration) featuring the same 6s^2^ lone pair, (2) shows a similar electronegativity (Bi: 2.02, Pb: 2.33, Sn: 1.96), and (3) has an ionic radius (103 pm) comparable to Pb^2+^ (119 pm) and Sn^2+^ (110 pm) metal cations [28, 51, 125].

However, the trivalent Bi^3+^ ion cannot directly replace the divalent Pb^2+^ ion in the perovskite structure due to the different valence state. Bismuth halide perovskites exhibit a huge structural diversity in terms of connectivity (face-, edge- or corner-sharing networks) and dimensionality ranging from zero-dimensional dimer units, to one-dimensional chain-like motifs or two-dimensional layered networks up to three-dimensional double perovskite frameworks (elpasolite structure) [66].

Zero-dimensional bismuth halide perovskites with a basic formula unit A_3_Bi_2_X_9_ crystallize in the Cs_3_Cr_2_Cl_9_ structure type. This crystal structure is based on the hexagonal closest packing of A and X atoms forming hexagonally stacked AX_3_ layers with trivalent metal cations occupying two-thirds of the emerging octahedral sites, while one-third of the remaining metal sites are vacant. In this way, double octahedral structures are obtained consisting of pairs of face-sharing BiX_6_ octahedra to give complex Bi_2_X_9_^3−^ anionic clusters, which are referred to as isolated metal halide dimer units. The resulting discrete anionic bi-octahedral moieties are surrounded by monovalent cations occupying the A-site of the perovskite structure [36, 66, 162, 164, 175–178]. Several zero-dimensional bismuth halide perovskites have been reported so far incorporating a range of different cations such as CH_3_NH_3_^+^ [36], guanidinium [161], cyclohexylammonium [179], K^+^ [66], Rb^+^ [66], or Cs^+^ [36, 66].

The most intensively studied bismuth halide perovskite in terms of optoelectronic applications is (CH_3_NH_3_)_3_Bi_2_I_9_. Single crystals can be synthesized via a layered-solution crystallization technique [176, 180], while thin films are obtained from solution-based processing (e.g. spin coating, doctor blading) followed by subsequent thermal annealing at low temperatures [36, 175, 181–184] or via vapor-assisted methods [181]. The (CH_3_NH_3_)_3_Bi_2_I_9_ structure consists of pairs of face-sharing BiI_6_ octahedra forming isolated metal halide dimer units of Bi_2_I_9_^3−^ surrounded by randomly disordered CH_3_NH_3_^+^ cations [175, 177, 180, 182]. The bi-octahedral anionic clusters are interconnected via N–H···I hydrogen bonding interactions [179, 182]. Dipolar ordering of the organic cation and in-plane ordering of the lone pair of the metal upon cooling is accompanied by phase transitions from a hexagonal crystal structure (space group: *P*6_3_/*mmc*) at 300 K to a monoclinic crystal structure (space group: *C*2/*c*) at 160 K with an additional first-order phase transition at 143 K (monoclinic, space group: *P*2_1_) [180].

(CH_3_NH_3_)_3_Bi_2_I_9_ is an environmentally friendly semiconductor with promising stability in ambient atmosphere and under humid conditions [36, 175, 180, 181, 183, 184]. With regard to the electronic band structure, DFT calculations predict an indirect character of the band gap with values of ca. 2.25 eV [175, 181], which are in good agreement with the experimental values (1.94-2.11 eV) [175, 176, 181]. In addition, (CH_3_NH_3_)_3_Bi_2_I_9_ exhibits a strong absorption band around 500 nm, a pre-edge absorption peak at 2.51 eV indicating the existence of intrinsic excitons, and a high optical absorption coefficient in the order of 10^5^ cm^−1^ comparable to that of lead-based analogues [36, 169, 176, 177, 184]. However, the exciton binding energy of more than 300 meV [177], which is in good agreement with time-dependent DFT calculations (400 meV) [182], is much larger than in lead-based analogues (ca. 40 meV [185]) and thus limits the photovoltaic performance up to now.

The potential of (CH_3_NH_3_)_3_Bi_2_I_9_ as lead-free absorber material for photovoltaic applications has been explored in planar [182, 184] and meso-structured [36, 175, 184] device configurations using diverse electron (e.g. TiO_2_ [36, 175, 183, 184], PCBM [182]) and hole (e.g. Spiro-OMeDAT [36, 183, 184], P3HT [175], PEDOT:PSS [182]) transport layers as well as the transparent conductive oxides FTO [36, 175, 183, 184] or ITO [182, 183].

**Fig. 12:**
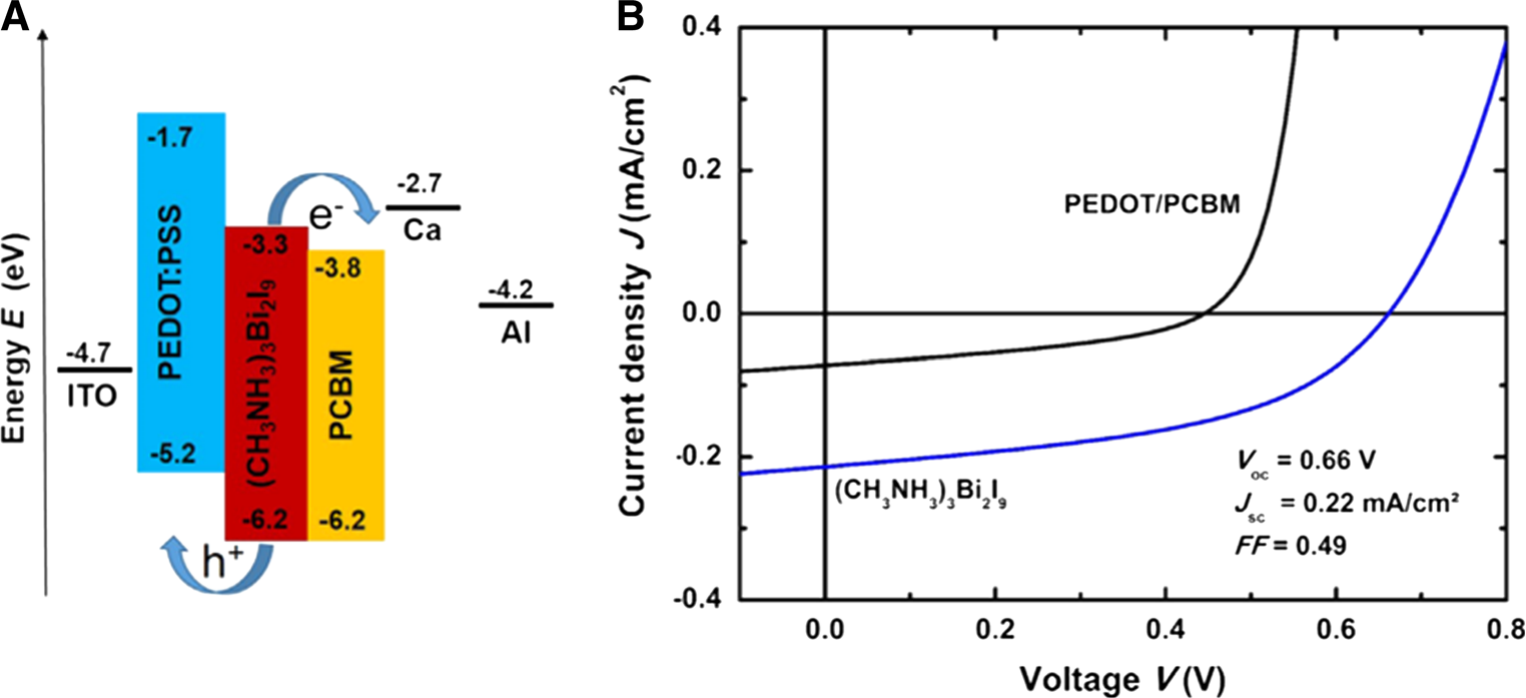
**a** Energy level diagram and **b** *J*–*V* curves under illumination of a photovoltaic device with a (CH_3_NH_3_)_3_Bi_2_I_9_-based absorber material (*blue*) and a reference solar cell without absorber (*black*). Adapted with permission from [182]. Copyright (2016) Elsevier

Öz et al. investigated (CH_3_NH_3_)_3_Bi_2_I_9_ in planar heterojunction solar cells in inverted geometry (ITO/PEDOT:PSS/(CH_3_NH_3_)_3_Bi_2_I_9_/PCBM/Ca/Al) and obtained a *V*_OC_ of 0.66 V, a FF of 49%, and a PCE of about 0.1% (Fig. 12) [182]. The photovoltaic performance is currently limited by the relatively low *J*_SC_ of 0.22 mA cm^−2^, which is due to the high exciton binding energy and ineffective charge separation in planar configurations [182].

In case of planar heterojunction solar cells with a general device architecture of FTO/TiO_2_/(CH_3_NH_3_)_3_Bi_2_I_9_/P3HT/Au a *V*_OC_ of 0.51 V, a *J*_SC_ of 0.36 mA cm^−2^, a FF of 44.4%, and a PCE of 0.08% could be achieved [175]. In perovskite solar cells (FTO/TiO_2_/mp-TiO_2_/perovskite/P3HT/Au) employing thick mesoporous TiO_2_ layers (1.8 µm), the photovoltaic performance can be improved yielding a *V*_OC_ of 0.35 V, a *J*_SC_ of 1.16 mA cm^−2^, a FF of 46.4%, and a PCE of ca. 0.19% [175].

Singh et al. evaluated the effect of various types of TiO_2_ (anatase, brookite) and architectures (planar, mesoporous) of ETLs on the film morphology and photovoltaic performance in solar cells (FTO/TiO_2_/(CH_3_NH_3_)_3_Bi_2_I_9_/Spiro-OMeDAT/Au) [184]. The implementation of a mesoporous anatase TiO_2_ scaffold was reported to significantly improve the *J*_SC_ (ca. 0.8 mA cm^−2^) and the efficiency (0.2%) compared to planar and mesoporous brookite perovskite solar cells. Almost no *J*–*V* hysteresis was determined irrespective of the type and architecture of the ETL. In addition, the solar cells were found to be moderately stable under ambient conditions without any sealing for more than 10 weeks [184].

**Fig. 13:**
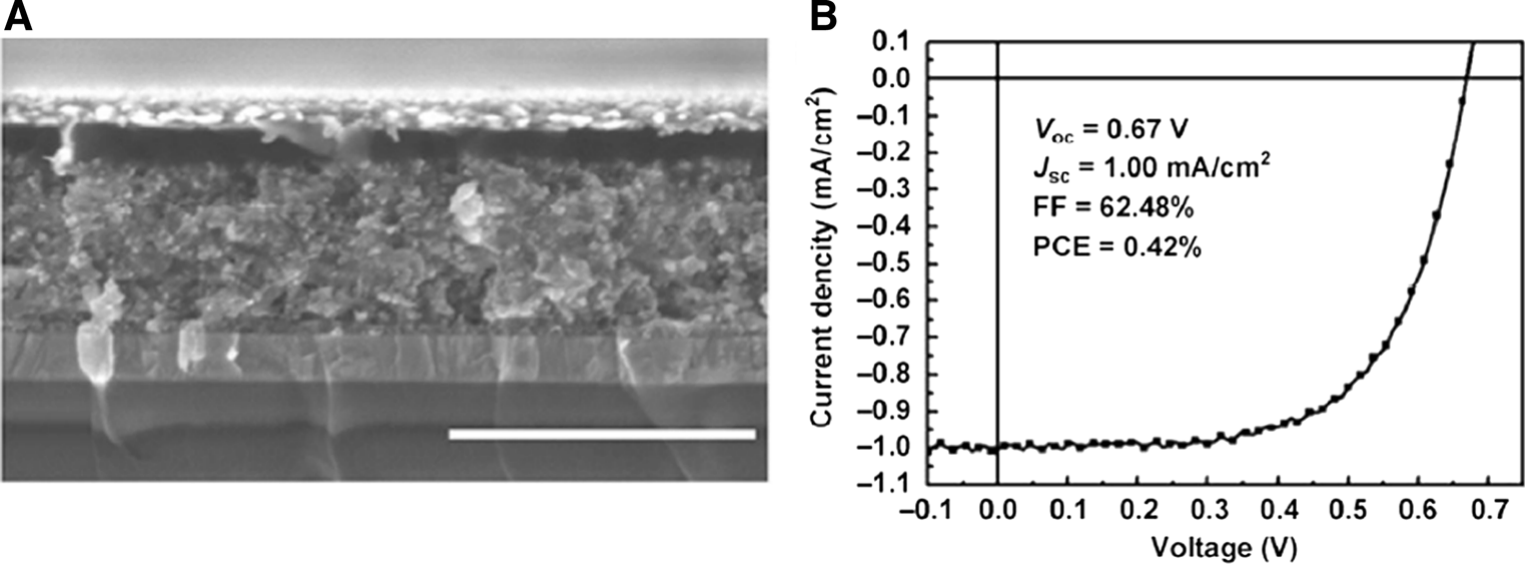
**a** Cross-sectional SEM image of a (CH_3_NH_3_)_3_Bi_2_I_9_-based perovskite solar cell in meso-structured configuration (ITO/c-TiO_2_/mp-TiO_2_/(CH_3_NH_3_)_3_Bi_2_I_9_/Spiro-OMeTAD/MoO_3_/Ag, *scale bar* 1 µm), **b** *J*–*V* curve under illumination (100 mW/cm^2^). Adapted with permission from [183]. Copyright (2016) Springer

Zhang et al. reported enhanced PCE values using ITO and a modified annealing procedure of the ETL instead of FTO as transparent contact [183]. In addition, the processing conditions and the structure of the ETL (planar or meso-structured) play a key role for the morphology of the active layer and consequently for the photovoltaic performance. The PCE was improved from 0.14% in planar architecture (ITO/c-TiO_2_/(CH_3_NH_3_)_3_Bi_2_I_9_/Spiro-OMeTAD/MoO_3_/Ag) to 0.42% in the meso-structured configuration (ITO/c-TiO_2_/mp-TiO_2_/(CH_3_NH_3_)_3_Bi_2_I_9_/Spiro-OMeTAD/MoO_3_/Ag, Fig. 13) [183].

**Fig. 14:**
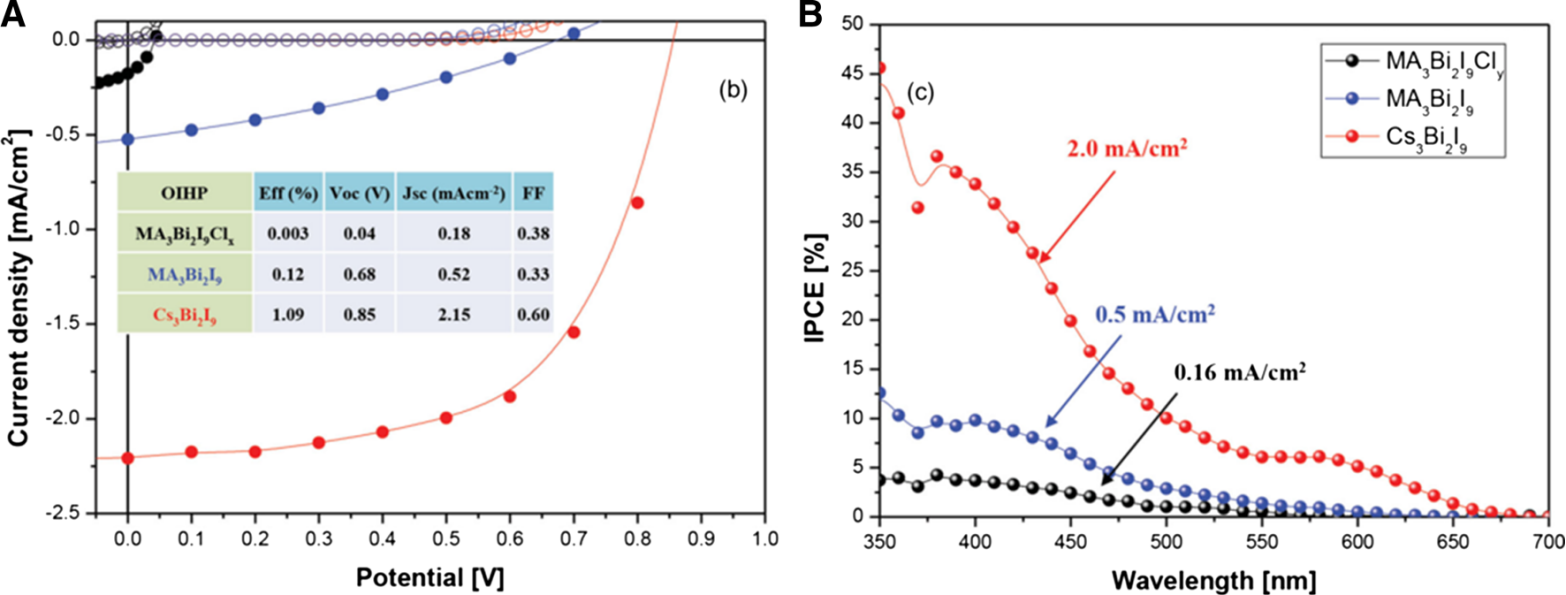
**a** *J*–*V* curves and **b** IPCE spectra of perovskite solar cells in meso-structured configuration using (CH_3_NH_3_)_3_Bi_2_I_9−*x*_Cl_*x*_, (CH_3_NH_3_)_3_Bi_2_I_9_, and Cs_3_Bi_2_I_9_ absorber materials, respectively. Adapted with permission from [36]. Copyright (2015) WILEY–VCH Verlag GmbH & Co. KGaA, Weinheim

Park et al. expanded the research to mixed halide pervoskites such as (CH_3_NH_3_)_3_Bi_2_I_9−*x*_Cl_*x*_ [36]. Due to the partial substitution of iodide with chloride in (CH_3_NH_3_)_3_Bi_2_I_9−*x*_Cl_*x*_, the band gap was shifted from 2.1 eV (X = I) to 2.4 eV (X = Cl, I) assuming a direct character of the band gap in both cases [36]. The photovoltaic performance in a meso-structured device architecture (FTO/c-TiO_2_/mp-TiO_2_/perovskite/Spiro-OMeDAT/Ag), however, was significantly lower (0.003%) compared to (CH_3_NH_3_)_3_Bi_2_I_9_ (0.12%), which can be attributed to the low *V*_OC_ of only 40 mV (Fig. 14) [36].

Moreover, the zero-dimensional dimer species of Cs_3_Bi_2_I_9_ was investigated previously with regard to the crystal structure and phase transitions [165, 178]. Recently, Cs_3_Bi_2_I_9_ has attracted substantial attention as alternative lead-free absorber for photovoltaic applications. Park et al. implemented Cs_3_Bi_2_I_9_ in meso-structured perovskite solar cells (FTO/c-TiO_2_/mp-TiO_2_/perovskite/Spiro-OMeDAT/Ag) and obtained a record efficiency of 1.09% for a bismuth halide perovskite solar cell (Fig. 14) [36]. Cs_3_Bi_2_I_9_ showed improved photovoltaic characteristics compared to the methylammonium analogue (Fig. 14a). In addition, while almost no *J*–*V* hysteresis was found directly after device fabrication, a pronounced hysteretic behavior was observed after a month. However, the PCE was shown to be highly stable with no decay even after storage under dry conditions during a month. Thus, Cs_3_Bi_2_I_9_ and other zero-dimensional analogues might be suitable candidates for solution-processed absorber materials to substitute lead-based perovskites.

One-dimensional bismuth halide perovskites exist in two different structures: (1) in form of BiX_4_^−^ anionic chains built of edge-sharing BiX_6_ octahedra alternating with cationic species to balance the charge neutrality (e.g. LiBiI_4_·5 H_2_O [186]) or (2) as bismuth halide chains of distorted BiX_6_ octahedra in zigzag conformation, which are interconnected by dicationic alkyldiammonium species occupying the A-site positions (e.g. HDABiI_5_ [54]).

The first motif can be found in LiBiI_4_·5 H_2_O, MgBi_2_I_8_·8 H_2_O, MnBi_2_I_8_·8 H_2_O, and KBiI_4_·H_2_O, which were studied by Yelovik et al. [186]. The optical band gaps of the four compounds were determined to be between 1.70 and 1.76 eV, which is in good agreement with the electronic band structure calculations for the KBiI_4_ model compound (1.78 eV). Due to these promising optical properties, one-dimensional perovskites might be prospective absorber materials for photovoltaic applications [186].

Fabian et al. investigated a one-dimensional bismuth halide perovskite based on corrugated metal halide chains of distorted corner-sharing BiI_6_ octahedra to give BiI_5_^2−^ units, which are interlinked via dicationic alkyldiammonium species [54]. The compound HDABiI_5_, with HDA = 1,6-hexanediammonium ([H_3_N-(CH_2_)_6_-NH_3_]^2+^), can be prepared via a solution-based method and crystallizes in an orthorhombic crystal structure [54, 187]. The optical band gap was determined to be 2.05 eV for an indirect transition. HDABiI_5_ was incorporated as absorber layer in meso-structured perovskite solar cells (FTO/c-TiO_2_/mp-TiO_2_/HDABiI_5_/Spiro-OMeTAD/Au) giving a *V*_OC_ of 0.40 V, a *J*_SC_ of 0.12 mA cm^−2^, a FF of 43%, and a PCE of 0.027% [54].

Two-dimensional layered structures are accommodated by metal-deficient or defect perovskites employing higher valent systems such as pnictogens, in which vacancies are present within the inorganic framework concomitant with trivalent metal cations. The crystal structure is based on a cubic close packing of A and X atoms with B-site cations occupying two-thirds of the octahedral cavities, while one-third of the remaining metal sites are vacant (K_3_Bi_2_I_9_ structure type). This results in the formation of inorganic metal-deficient layers of the type B_2/3_X_4_^2−^, which are built up of corrugated layers of corner-sharing, distorted BX_6_ octahedra to give a two-dimensional structure. The structure can be, therefore, considered as distorted defect variant of the classical three-dimensional ABX_3_-type perovskite [66].

K_3_Bi_2_I_9_ and Rb_3_Bi_2_I_9_ are examples for two-dimensional layered defect perovskites. Both compounds can be prepared by solution-based or solid-state reactions, and were shown to exhibit an improved stability under ambient conditions compared to lead- and tin-based analogues [66]. The optical band gaps were determined to be 2.1 eV for both compounds with a direct band character as predicted from electronic band structure calculations [66]. In contrast to that, the Cs_3_Bi_2_I_9_ analogue with the larger A-site cation Cs^+^ can only adopt a zero-dimensional perovskite structure with totally different optoelectronic properties as discussed before.

However, recently Johansson et al. reported on a layered perovskite structure for CsBi_3_I_10_, which was prepared via a solution-based processing method by adjusting the stoichiometric composition of the starting materials CsI and BiI_3_ [188]. CsBi_3_I_10_ features a layered structure similar to BiI_3_ alternating with zero-dimensional structures as found in Cs_3_Bi_2_I_9_. CsBi_3_I_10_ exhibits a band gap of 1.77 eV similar to BiI_3_ and an absorption coefficient of 1.4 × 10^5^ cm^−1^, which is comparable to lead-based analogues [169, 188]. In comparison to the zero-dimensional Cs_3_Bi_2_I_9_ compound (2.03 eV), the layered CsBi_3_I_10_ has a lower band gap, which results in improved light-harvesting properties. In addition, CsBi_3_I_10_ shows improved film formation properties compared to Cs_3_Bi_2_I_9_ with more uniform, smoother and pinhole-free layers, which is advantageous for photovoltaic applications. CsBi_3_I_10_ was implemented as absorber material in meso-structured solar cells (FTO/c-TiO_2_/mp-TiO_2_/perovskite/P3HT/Ag) yielding a PCE of 0.40%, which is significantly higher compared to the Cs_3_Bi_2_I_9_ (0.02%) and BiI_3_ (0.07%) solar cells obtained in the same device architecture [188] but significant lower compared to the Cs_3_Bi_2_I_9_-based solar cells obtained by Park et al. (PCE of 1.09%) [36].

Another example for a two-dimensional layered perovskite structure is (NH_4_)_3_Bi_2_I_9_ [48, 189]. (NH_4_)_3_Bi_2_I_9_ crystallizes in a monoclinic crystal system [189] and has a similar structure as the Rb and K analogues discussed above. Hydrogen bonding interactions of the type N–H···I were found to be essential for the stabilization of the layered structure [189]. Besides the low-temperature solution processability, (NH_4_)_3_Bi_2_I_9_ has an optical band gap of 2.04 eV, which is comparable to the band gaps of the above-discussed Rb and K analogues (2.1 eV). A further example for a layered perovskite structure is the metal-deficient (H_2_AEQT)B_2/3_I_4_ (B = Sb, Bi) perovskite, where AEQT is 5,5′′′-bis-(aminoethyl)-2,2′:5′,2′′:5′′,2′′′-quaterthiophene [57]. However, both (NH_4_)_3_Bi_2_I_9_ and (H_2_AEQT)B_2/3_I_4_ have not been used as absorber material for photovoltaic applications so far.

**Fig. 15:**
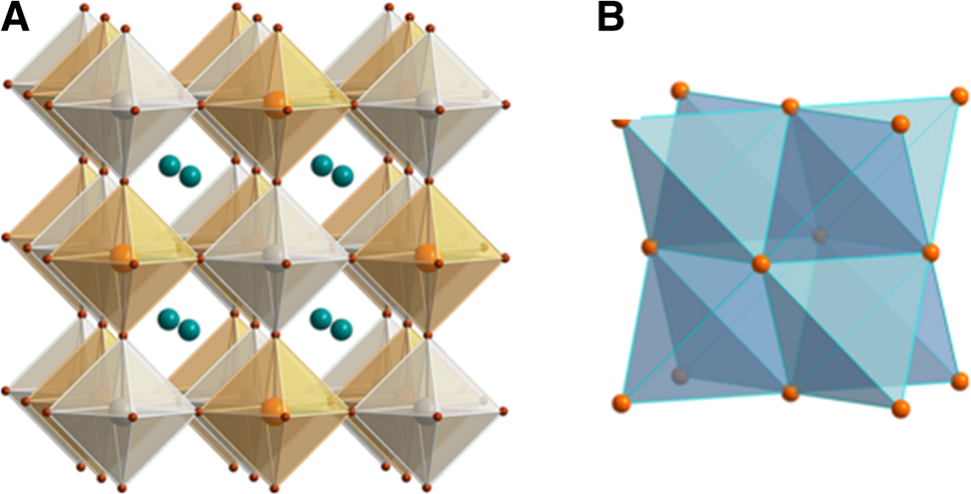
**a** Crystal structure of rock-salt ordered double halide perovskites (turquoise: monovalent A-site cation,* gray* monovalent B^I^ cation,* orange* trivalent B^II^ cation,* brown* halide counterion). **b** Face-centered cubic sublattice in double halide perovskites comprising edge-sharing tetrahedral positions. Adapted with permission from [64]. Copyright (2016) American Chemical Society

Three-dimensional perovskite structures containing bismuth have only been obtained in quaternary double perovskites with a basic formula unit of A_2_B^I^B^II^X_6_ [16, 58, 60, 150] by heterovalent substitution of Pb^2+^ by a combination of a monovalent Bi^+^ (B^I^) and a trivalent Bi^3+^ (B^II^) cation. The double perovskite structure (elpasolite) is based on corner-sharing B^I^X_6_ and B^II^X_6_ octahedra alternating along the three crystallographic axes in a rock-salt ordered cubic structure to form a three-dimensional network with mono- and trivalent metal ions occupying the B^I^ and B^II^ sites, respectively [16, 63, 64, 190]. The cuboctahedral cavities within this elpasolite structure are occupied by A-site cations such as Cs^+^ or CH_3_NH_3_^+^ (Fig. 15) [16, 190, 191].

Such quaternary halide double perovskite structures can be found for mixed-valent perovskite systems based on thallium (e.g. Cs_2_Tl^+^Tl^3+^X_6_ (X = F, Cl) [58]) and gold (e.g. Cs_2_Au^+^Au^3+^I_6_ [61]) as well. Other examples of halide double perovskites are based on monovalent alkali metal (e.g. Na^+^) and noble-metal (e.g. Cu^+^, Ag^+^, Au^+^) cations and trivalent metal ions such as group-13 elements (e.g. In^3+^, Tl^3+^), pnictogens (e.g. Sb^3+^, Bi^3+^), lanthanides (e.g. La^3+^, Ce^3+^, Pr^3+^, Nd^3+^, Sm^3+^, Eu^3+^, Gd^3+^, Dy^3+^, Er^3+^, Tm^3+^, Lu^3+^), and actinides (e.g. Pu^3+^, Am^3+^, Bk^3+^) [63, 150]. Considering bismuth-based halide double perovskites, various compounds have been investigated with regard to the synthesis and crystal structure as well as optical and electronic properties in theory and experiment [16, 63, 64, 190, 191]. Cs_2_AgBiX_6_ (X = Cl, Br) perovskites, for example, can be synthesized via a solution-based or a solid-state reaction, crystallize in the elpasolite structure, and exhibit improved stability in terms of heat and moisture under ambient conditions compared to lead-based halide perovskites [16, 64, 190]. However, Cs_2_AgBiBr_6_ was still found to degrade upon exposure to air and light over a period of weeks [16]. Cs_2_AgBiCl_6_ and Cs_2_AgBiBr_6_, are indirect semiconductors with experimental band gaps in the range of 2.2–2.77 eV for Cs_2_AgBiCl_6_ and 1.95–2.19 eV for Cs_2_AgBiBr_6_ [16, 63, 64, 190].

The family of pnictogen-noble metal halide double perovskites is especially interesting for photovoltaic applications because of the structural similarity, i.e. three-dimensional structure, to lead-based perovskites despite the different valence of the metal cations incorporated. In addition, a huge variety of material compositions is amenable due to the high number of possible element combinations of monovalent (B^I^ = Cu^+^, Ag^+^, Au^+^) and trivalent (B^II^ = Sb^3+^, Bi^3+^) metal cations together with organic and inorganic cations (A) and halide anions (X). Based on first-principle calculations, pnictogen-noble metal halide double perovskites have low carrier effective masses, and the calculated electronic band gaps were found to be tunable in the visible range depending on the choice of the noble metal, i.e. 1.3–2.0 eV (Cs_2_CuBiX_6_), 1.6–2.7 eV (Cs_2_AgBiX_6_), and 0.5–1.6 eV (Cs_2_AuBiX_6_) [63].

Hybrid halide double perovskites such as (CH_3_NH_3_)_2_KBiCl_6_ incorporating organic cations have been reported recently [191]. (CH_3_NH_3_)_2_KBiCl_6_ was prepared using a hydrothermal method through the reaction between CH_3_NH_3_Cl, KCl, and BiCl_3_. Theoretical calculations of the electronic structure predict an indirect character of the band gap (3.02 eV), which is in good agreement with the experimental value of 3.04 eV determined from reflectance measurements and comparable to the lead analogue CH_3_NH_3_PbCl_3_ (2.88 eV [191, 192]). However, no solar cell data have been reported yet.

Structural, optical as well as solar cell data of bismuth halide perovskites are summarized in Table 9.

**Table 9:** Structural and optical data of bismuth halide perovskites and the highest obtained PCEs (if applied in photovoltaic devices)

Perovskite	Sim./exp.	Crystal system (space group)	Dimensionality	Band gap/eV	PCE/%	References
(NH_4_)_3_Bi_2_I_9_	Sim./exp.	Monoclinic (*P*2_1_/*c*)	2D	2.04	–	[189]
(CH_3_NH_3_)_3_Bi_2_Br_9_	Exp.	Trigonal (*P* $\bar{3}$ *m*1)	–	–	–	[158]
(CH_3_NH_3_)_3_Bi_2_I_9_	Sim./exp.	Hexagonal (*P*6_3_/*mmc*) at 300 K	0D dimer	1.94–2.11	0.42	[36, 175–177, 180, 182–184, 193]
		Monoclinic (*C*2/*c*) at 160 K		2.04		[180, 181]
		Monoclinic (*P*2_1_) at 100 K		–		[180]
(CH_3_NH_3_)_3_Bi_2_I_9−*x*_Cl_*x*_	Exp.	Hexagonal (*P*6_3_/*mmc*)	–	2.4	0.003	[36]
(C_6_H_14_N)_3_Bi_2_I_9_	Sim./exp.	Monoclinic (*Pc*)	0D dimer	2.9	–	[179]
K_3_Bi_2_I_9_	Sim./exp.	Monoclinic (*P*2_1_/*n*)	2D	2.1	–	[66]
Rb_3_Bi_2_Br_9_	Exp.	Orthorhombic (*Pnma*)	–	2.62	–	[162]
Rb_3_Bi_2_I_9_	Sim./exp.	Monoclinic (*Pc*)	2D	1.89–2.1	–	[162]
		Monoclinic (*P*2_1_/*n*)				[66]
Cs_3_Bi_2_Br_9_	Exp.	Trigonal (*P* $\bar{3}$ *m*1)	2D	2.50	–	[162]
Cs_3_Bi_2_I_9_	Sim./exp.	Hexagonal (*P*6_3_/*mmc*)	0D dimer	1.8–2.2	1.09	[16, 36, 66, 162, 164]
(CH_3_NH_3_)_2_KBiCl_6_	Sim./exp.	Trigonal (*R* $\bar{3}$ *m*)	3D	3.04	–	[191]
Cs_2_CuBiX_6_ (X = Cl, Br, I)	Sim.	Cubic (*Fm* $\bar{3}$ *m*)	3D	2.0 (X = Cl)	–	[63]
				1.9 (X = Br)		
				1.3 (X = I)		
Cs_2_AgBiCl_6_	Sim./exp.	Cubic (*Fm* $\bar{3}$ *m*)	3D	2.2–2.77	–	[16, 63, 190]
Cs_2_AgBiBr_6_	Sim./exp.	Cubic (*Fm* $\bar{3}$ *m*)	3D	1.95–2.19	–	[16, 63, 64, 190]
Cs_2_AgBiI_6_	Sim.	Cubic (*Fm* $\bar{3}$ *m*)	3D	1.6	–	[63]
Cs_2_AuBiX_6_ (X = Cl, Br, I)	Sim.	Cubic (*Fm* $\bar{3}$ *m*)	3D	1.6 (X = Cl)	–	[63]
				1.1 (X = Br)		
				0.5 (X = I)		
LiBiI_4_ 5 H_2_O	Exp.	Monoclinic (*C*2/*c*)	1D	1.7–1.76	–	[186]
MgBi_2_I_8_·8 H_2_O	Exp.	Monoclinic (*P*2_1_/*c*)	1D	1.7–1.76	–	[186]
MnBi_2_I_8_·8 H_2_O	Exp.	Monoclinic (*P*2_1_/*c*)	1D	1.7–1.76	–	[186]
KBiI_4_·H_2_O	Exp.	Monoclinic (*P*2_1_/*n*)	1D	1.7–1.76	–	[186]
HDABiI_5_	Exp.	Orthorhombic	1D	2.05	0.027	[54, 187]
[C(NH_2_)_3_]_3_Bi_2_I_9_	Exp.	Orthorhombic (*Cmcm*)	–	–	–	[161]
(C_10_H_7_NH_3_)BiI_4_	Exp.	Orthorhombic (*Pbca*)	1D	2.32	–	[194]
[C_6_H_4_(NH_3_)_2_]_2_Bi_2_I_10_·4 H_2_O	Exp.	Monoclinic (*P*2_1_/*n*)	0D	2.84	–	[195]
(H_2_AEQT)Bi_2/3_I_4_	Exp.	Monoclinic (*C*2/*m*)	2D	–	–	[173]
CsBi_3_I_10_	Exp.	–	2D	1.77	0.40	[188]

### Tellurium halide perovskites

Tellurium is a group-16 element with relatively low abundance in the Earth’s crust. There are various aspects that suggest tellurium as potential heterovalent replacement candidate for lead in the perovskite structure. The tetravalent Te^4+^ cation (1) is isoelectronic to Sn^2+^ (4d^10^ 5s^2^) and has a similar s^2^ valence electronic configuration as the divalent Pb^2+^ featuring a 5s^2^ lone pair, (2) has a comparable electronegativity (Te: 2.1, Sn: 1.96, Pb: 2.33) but (3) a slightly smaller ionic radius (97 pm) compared to the divalent Sn^2+^ (110 pm) and Pb^2+^ (119 pm) metal cations [51, 125].

Tellurium halide perovskites with the general formula A_2_TeX_6_ employing ammonia (NH_4_^+^), alkali metal cations (K^+^, Rb^+^, Cs^+^), and thallium (Tl^+^) as A-site cation and halide counterions (Cl^−^, Br^−^, I^−^) were investigated with regard to crystal structure, optical and other physicochemical properties [162, 196]. The inorganic tellurium iodide perovskites A_2_TeI_6_ (A = K, Rb, Cs, Tl) are especially interesting for photovoltaic applications due to the low band gaps in the range of 1.38–1.52 eV [162]. Cs_2_TeI_6_, for example, was investigated by Maughan et al. [96]. The crystal structure of this compound is derived from the three-dimensional double perovskite structure (A_2_B^I^B^II^X_6_). While one B-site (B^I^) is accommodated by the tetravalent tellurium cation, the other one (B^II^) is replaced with a vacancy forming a vacancy-ordered cubic double perovskite of the type A_2_BX_6_ (K_2_PtCl_6_ structure type), in which discrete BX_6_^2−^ octahedra are interconnected by monovalent A-site cations occupying the cuboctahedral voids [96]. Electronic band structure calculations indicate an indirect band gap. The experimental band gap was determined to be between 1.52 and 1.59 eV [96, 162]. A summary of structural and optical data of tellurium halide perovskites is given in Table 10. However, to the best of our knowledge, tellurium-based perovskites have not been examined as alternative lead-free absorber material for photovoltaics.

**Table 10:** Structural and optical data of tellurium halide perovskites. Dimensionalities and PCE values have not been reported

Perovskite	Sim./exp.	Crystal system (space group)	Band gap/eV	References
(NH_4_)_2_TeCl_6_	Exp.	Cubic (*Fm* $\bar{3}$ *m*)	–	[196]
K_2_TeCl_6_	Exp.	Monoclinic (*P*2_1_/n)	–	[196]
Rb_2_TeCl_6_	Exp.	Cubic (*Fm* $\bar{3}$ *m*)	–	[196]
Cs_2_TeCl_6_	Exp.	Cubic (*Fm* $\bar{3}$ *m*)	–	[196]
(NH_4_)_2_TeBr_6_	Exp.	Cubic (*Fm* $\bar{3}$ *m*)	–	[196]
K_2_TeBr_6_	Exp.	Monoclinic (*P*2_1_/*c*)	2.17	[162, 196]
Rb_2_TeBr_6_	Exp.	Cubic (*Fm* $\bar{3}$ *m*)	2.19	[162]
Cs_2_TeBr_6_	Exp.	Cubic (*Fm* $\bar{3}$ *m*)	2.20	[162]
Tl_2_TeBr_6_	Exp.	Tetragonal (*P*4/*mnc*)	2.06	[162]
(NH_4_)_2_TeI_6_	Exp.	Monoclinic (*P*2_1_/n)	–	[196]
K_2_TeI_6_	Exp.	Monoclinic (*P*2_1_/*c*)	1.38	[162]
Rb_2_TeI_6_	Exp.	Tetragonal (*P*4/*mnc*)	1.43	[162, 196]
Cs_2_TeI_6_	Exp.	Cubic (*Fm* $\bar{3}$ *m*)	1.52–1.59	[96, 162]
Tl_2_TeI_6_	Exp.	Monoclinic (*P*2_1_/*c*)	1.47	[162]

## Mixed metal halide-chalcogenide and metal chalcogenide perovskites

Even though much progress has been made in the field of alternative lead-free perovskite semiconductors and many new absorber materials for photovoltaic applications have been proposed, these new materials have been shown to be not fully competitive in terms of efficiency and they suffer from problems such as chemical stability and toxicity, which are still not fully overcome. However, it is also possible to introduce chalcogenide anions into the perovskite structure by replacing the halides partly or fully.

In a first approach, the split-anion method is based on the partial substitution of halide with chalcogenide anions in ABX_3_-type metal halide perovskites forming mixed chalcogenide-halide perovskites with a general formula AB(Ch,X)_3_ [197]. Due to the more covalent bonding character of metal–chalcogenide bonds compared to metal halide bonds, mixed chalcogenide-halide compounds are proposed to exhibit an enhanced stability under ambient atmosphere [198].

**Fig. 16:**
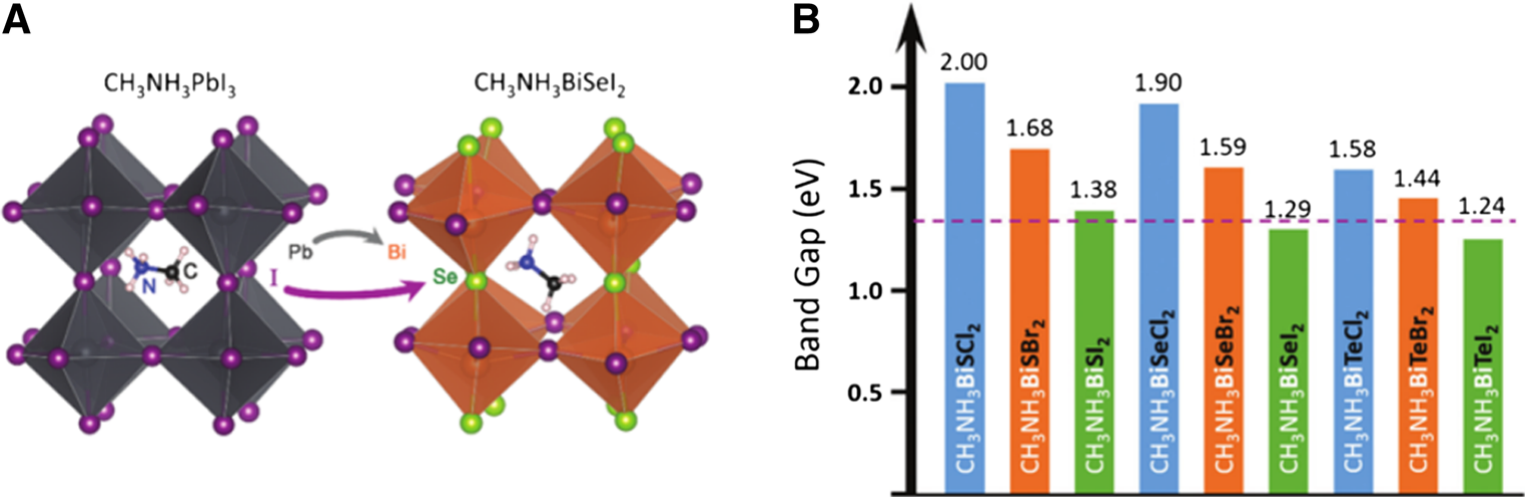
**a** Atomic structures of CH_3_NH_3_PbI_3_ and CH_3_NH_3_BiSeI_2_, and schematic representation of the split-anion approach for the replacement of Pb in CH_3_NH_3_PbI_3_; **b** Calculated band gaps of CH_3_NH_3_BiXY_2_ (X = S, Se, Te; Y = Cl, Br, I) using HSE functional with spin–orbit coupling. The *dashed line* indicates the optimal band gap for single-junction solar cells according to the Shockley–Queisser theory. Adapted with permission from [197]. Copyright (2016) Royal Society of Chemistry

Sun et al. theoretically investigated the potential of the split-anion approach for bismuth-based perovskites using first-principles calculations [197]. The halogen anions (X = Cl, Br, I) are partially substituted with chalcogenides (Ch = S, Se, Te), i.e. one per formula unit, to obtain I–III–VI–VII_2_-type semiconductors with the formula CH_3_NH_3_BiChX_2_ [197] exhibiting calculated direct band gaps in the range of 1.24–2.00 eV (Fig. 16). CH_3_NH_3_BiSeI_2_ and CH_3_NH_3_BiSI_2_, in particular, were identified as promising absorber materials with direct band gaps of 1.3 and 1.4 eV, respectively [197].

Hong et al. investigated inorganic mixed-anion perovskites with a general AB(Ch,X)_3_ structure (A = Cs, Ba; B = Sb, Bi; Ch = chalcogen; X = halogen), where halogen anions are partially replaced with chalcogenide anions [198]. According to DFT calculations, the examined perovskite materials were found to be thermodynamically unstable and to decompose into secondary phases [198]. This instability was supported in solid-state synthesis experiments by the formation of distinct halide and chalcogenide phases or mixed-anion phases with non-perovskite structures [198]. Sun et al. theoretically examined CsSnS_2_Cl as an example for an inorganic mixed-anion perovskite as prospective candidate as light absorber for photovoltaic applications [197]. Hybrid functional calculations estimated an indirect band gap of ca. 1 eV for CsSnS_2_Cl in the distorted perovskite phase and predicted promising optical absorption properties even higher than for CsSnI_3_ [197].

Up to now, the mixed chalcogenide-halide approach has not yielded new absorber materials but the huge variety of possible element compositions for new I–III–VI–VII_2_, II–II–VI–VII_2_, I–IV–VI_2_–VII or II–III–VI_2_–VII semiconductors makes the split-anion approach interesting for further research.

Improved stability properties can be expected in the case of total substitution of halide with chalcogenide anions [198]. This leads to a class of metal chalcogenide perovskite (ABCh_3_) semiconductors, which have already been studied back in the 1950s [199]. Recently, this class has come into the focus as potential absorber materials for photovoltaic applications [197, 198, 200, 201].

**Fig. 17:**
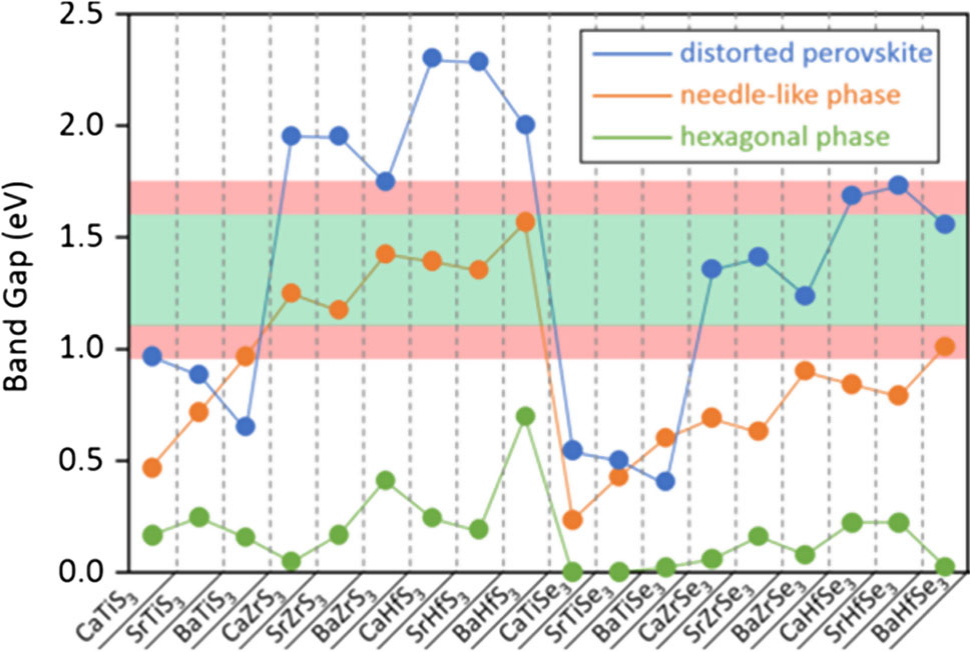
Calculated band gaps of 18 ABX_3_ compounds in the *distorted*, *hexagonal*, and *needle*-*like* phase using HSE06 functional. The optimal band gap region for solar cells is highlighted in *green*, while an extended region is highlighted in *light red*. Adapted with permission from [201]. Copyright (2015) American Chemical Society

DFT calculations of metal chalcogenide perovskites (ABCh_3_) with group-2 alkaline-earth metal cations (A = Ca^2+^, Sr^2+^, Ba^2+^), tetravalent group-4 metal cations (B = Ti^4+^, Zr^4+^, Hf^4+^), and chalcogenide (Ch = S^2−^, Se^2−^) ions predict promising band gaps and absorption behavior for CaTiS_3_, BaZrS_3_, CaZrSe_3_, and CaHfSe_3_ in the distorted perovskite phase [201]. For example, a direct band gap of 1.35 eV was calculated for CaZrSe_3_ [201]. Figure 17 displays the calculated values for these ABCh_3_ perovskite assuming three different structural motifs, a distorted perovskite phase, a needle like structure and a hexagonal structure.

Sun et al. theoretically investigated the substitution of Ba^2+^ in BaZrS_3_ with hydrazinium ((NH_3_NH_3_)^2+^) as molecular cation forming (NH_3_NH_3_)ZrS_3_ compounds [201]. DFT calculations predicted a direct band gap of 1.68 eV.

Wang et al. extended the DFT studies to the family of metal sulfide perovskites with three-dimensional ABS_3_ structure to two-dimensional, layered Ruddlesden–Popper perovskite sulfides A_3_B_2_S_7_, where A are alkaline-earth metals and B are transition metals [202]. Based on the layered structure, the formula can be also expressed as AS[ABS_3_]_*n*_ (*n* = 2), where ABS_3_ perovskite units alternate with additional AS layers for every *n* perovskite unit. This class of materials was reported to show a semiconducting ferroelectric photovoltaic behavior, i.e. photo-generated electron–hole pairs can be separated efficiently due to a stable ferroelectric polarization, and first-principles calculations predicted direct band gaps in the range of 1.8–2.4 eV [202].

Various metal chalcogenide perovskites have been investigated extensively with regard to the crystal structures and physicochemical properties in the last decades [199, 203–208]. Perera et al., for example, prepared chalcogenide perovskites such as SrTiS_3_, CaZrS_3_, SrZrS_3_, and BaZrS_3_ by high-temperature sulfurization of oxide perovskite analogues with carbon disulfide [200]. BaZrS_3_ and CaZrS_3_ exhibited direct band gaps of 1.73 and 1.90 eV, respectively, which were determined via UV–Vis and photoluminescence measurements, making them to potential absorber materials for photovoltaic applications [200]. In addition, the band gap was shown to be widely tunable using an anion alloying approach, i.e. engineering of the composition of metal chalcogenides based on the (partial) substitution of chalcogenide anions [200]. Using BaZrS_3_ as an example, the composition can be tuned systematically by partial substitution of the sulfide ion with oxygen ions under formation of transition metal oxysulfide perovskites BaZr(O_*x*_S_1−*x*_)_3_ exhibiting band gaps over a wide range from 1.73 eV in case of BaZrS_3_ to 2.87 eV for oxysulfide perovskites [200]. Moreover, the examined transition metal chalcogenide perovskite materials showed improved chemical stability under ambient atmosphere compared to metal halide perovskite analogues, which is due to the more covalent bonding character of the metal–chalcogenide bond [198, 200]. In addition, oxidic perovskites might become interesting for photovoltaic applications in the future and some materials with suited optical properties (e.g. BiFeO_3_ [209–211]) have already been investigated in photovoltaic devices.

## Conclusion

Among all reported lead-free perovskite materials, tin-based perovskites have been most intensively investigated up to now and show the highest PCE values of all alternative perovskite solar cells. PCE values of approximately 6% have been obtained with CH_3_NH_3_SnI_3_ and CH(NH_2_)_2_SnI_3_. Even though the stability of tin perovskites is lower compared to lead-based perovskite absorbers, progress has been made on this topic and a lifetime test over 77 days without an efficiency decay has already been reported. This makes tin-based perovskites to very promising materials for the realization of low-cost and sustainable lead-free solar cells. Germanium halide perovskites have very similar band gaps to lead-based compounds. However, they are chemically more unstable and much less investigated than tin-based perovskites, which is maybe also the reason why the PCEs of germanium perovskite-based solar cells remained significantly lower so far.

Alkaline-earth metals such as magnesium, calcium, strontium, and barium are suitable candidates for homovalent substitution of lead in the perovskite structure due to ionic radii comparable to lead. Magnesium iodide perovskites, in particular, were shown to have a tunable band gap in the visible range (0.9–1.7 eV [49]). Calcium-, strontium-, and barium-based halide perovskites, however, are possibly not a good alternative to lead halide perovskite semiconductors for photovoltaic applications due to the high band gaps (2.95–3.6 eV [118]), and their sensitivity towards humidity [118].

In addition, the family of transition metal-based halide perovskites, which often feature lower dimensional structures isostructural to Ruddlesden–Popper phases arising from the smaller ionic radii of the respective transition metals, has attracted considerable attention. Copper halide perovskites, in particular, are among the most promising transition metal-based perovskites with PCEs up to 0.63% [34].

Antimony halide perovskites are a further emerging class of lead-free semiconductors with promising optoelectronic properties. A key aspect of antimony halide perovskites is the enormous structural diversity ranging from zero-dimensional dimer to three-dimensional elpasolite-type double halide perovskite structures, which can not only be manipulated by the nature and size of the cationic and anionic species but also by the processing methodology [65]. For antimony-based perovskite solar cells, PCE values up to 0.66% are reported [35]. However, research on this material for photovoltaic applications is still in the beginning and rapid progress in terms of performance as well as in the development of interesting alternative perovskite-type semiconductors is expected.

The huge structural diversity ranging from zero-dimensional up to three-dimensional structures together with tunable band gaps in the visible range makes also bismuth halide perovskites a promising alternative with PCE values already exceeding 1% [36]. Bismuth perovskites show improved environmental stability compared to tin- or germanium-based perovskites.

Moreover, metal chalcogenide perovskite semiconductors provide a promising solution to address the limited chemical instability and the toxicity issue of lead-based systems. New strategies in materials design and band gap engineering over a wide range by tuning the stoichiometry and compositions, for example via a split-anion or an anion alloying approach to form mixed halide-chalcogenide compounds, enable the development of a remarkable number of novel absorber materials. Theoretical calculations predicting promising direct band gaps and improved optical absorption properties within the visible range compared to lead-based analogues highlight the potential of metal chalcogenide perovskite semiconductors for photovoltaics.
